# Genetic spectrum of Charcot–Marie–Tooth disease associated with myelin protein zero gene variants in Japan

**DOI:** 10.1111/cge.13881

**Published:** 2020-11-27

**Authors:** Takaki Taniguchi, Masahiro Ando, Yuji Okamoto, Akiko Yoshimura, Yujiro Higuchi, Akihiro Hashiguchi, Kensuke Shiga, Arisa Hayashida, Taku Hatano, Hiroyuki Ishiura, Jun Mitsui, Nobutaka Hattori, Toshiki Mizuno, Masanori Nakagawa, Shoji Tsuji, Hiroshi Takashima

**Affiliations:** ^1^ Department of Neurology and Geriatrics Kagoshima University Graduate School of Medical and Dental Sciences Kagoshima Japan; ^2^ Department of Physical Therapy, School of Health Sciences, Faculty of Medicine Kagoshima University Kagoshima Japan; ^3^ Department of Neurology Matsushita Memorial Hospital Osaka Japan; ^4^ Department of Neurology Juntendo University School of Medicine Tokyo Japan; ^5^ Department of Molecular Neurology, Graduate School of Medicine The University of Tokyo Tokyo Japan; ^6^ Department of Neurology Kyoto prefectural University of Medicine Kyoto Japan; ^7^ North Medical Center Kyoto prefectural University of Medicine Kyoto Japan; ^8^ Institute of Medical Genomics International University of Health and Welfare Chiba Japan

**Keywords:** cerebrospinal fluid protein, Charcot–Marie–Tooth disease, cranial nerve involvement, creatine kinase, myelin P0 protein

## Abstract

We aimed to reveal the genetic features associated with *MPZ* variants in Japan. From April 2007 to August 2017, 64 patients with 23 reported *MPZ* variants and 21 patients with 17 novel *MPZ* variants were investigated retrospectively. Variation in *MPZ* variants and the pathogenicity of novel variants was examined according to the American College of Medical Genetics standards and guidelines. Age of onset, cranial nerve involvement, serum creatine kinase (CK), and cerebrospinal fluid (CSF) protein were also analyzed. We identified 64 CMT patients with reported *MPZ* variants. The common variants observed in Japan were different from those observed in other countries. We identified 11 novel pathogenic variants from 13 patients. Six novel *MPZ* variants in eight patients were classified as likely benign or uncertain significance. Cranial nerve involvement was confirmed in 20 patients. Of 30 patients in whom serum CK levels were evaluated, eight had elevated levels. Most of the patients had age of onset >20 years. In another subset of 30 patients, 18 had elevated CSF protein levels; four of these patients had spinal diseases and two had enlarged nerve root or cauda equina. Our results suggest genetic diversity across patients with *MPZ* variants.

## Graphical abstract

Genetic spectrum of MPZ variants was confirmed in this study.

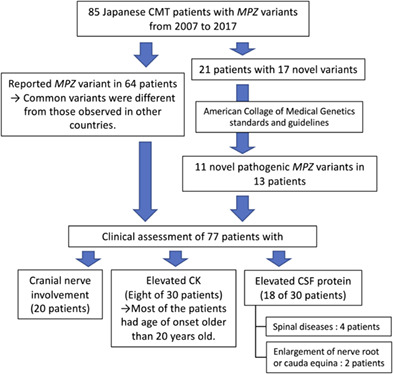

## 1 INTRODUCTION

Myelin protein zero (MPZ) protein is a major structural component of myelin and encoded by *MPZ* gene, which is expressed by Schwann cells.^1^ MPZ protein is classified as a member of immunoglobulin superfamily and an essential membrane protein comprising 248 amino acids.^2^ The final structure of MPZ protein consists of three domains: extracellular domain comprising 124 amino acids, transmembrane domain comprising 26 amino acids, and intracellular domain comprising 69 amino acids located at the C‐terminus.^3^, ^4^

Charcot–Marie–Tooth disease (CMT) is the most common inherited peripheral neuropathy. CMT is commonly divided into two groups: demyelinating type with slower median nerve conduction velocity (<38 m/s) and axonal type with maintained median nerve conduction velocity (>38 m/s).^5^

*MPZ* variants contribute to the cause of demyelinating neuropathy CMT1B (OMIM 118200) or axonal neuropathy CMT2I/J (OMIM 607677/607736) and also the more severe, juvenile‐onset Dejerine‐Sottas syndrome (OMIM 145900) and hypomyelinating neuropathy, congenital, 2 (OMIM 618184).^1^, ^6^ Moreover, *MPZ* variants are associated with dominant intermediate Charcot–Marie–Tooth disease D (CMTDID) (OMIM 607791).^7^ The phenotype of CMT caused by *MPZ* variants varies from severe pediatric onset to mild adult onset.^1^

To date, about 250 variants of this gene have been described as the cause of inherited peripheral neuropathy (https://portal.biobase-international.com/hgmd/pro/). There are limited studies that analyzed large number of patients with *MPZ* variants.^6^, ^8^ Our laboratory analyzed the genetic spectrum of Japanese patients with CMT.^9^

In this study, we investigated 85 patients to clarify the genetic spectrum of inherited peripheral neuropathy associated with *MPZ* variants in Japan. In addition, we also investigated the age of onset, cranial nerve involvement, serum creatine kinase (CK), and cerebrospinal fluid (CSF) protein in 77 patients with reported and novel pathogenic variants.

## 2 MATERIALS AND METHODS

### 2.1 Subjects

We examined 1657 Japanese patients who were considered to have inherited peripheral neuropathy from April 2007 to August 2017. All patients and family members provided written informed consent to participate in the study. Before starting this study, patients suspected to have demyelinating CMT with median motor nerve conduction velocity (median MCV) below 38 m/s were checked for duplication or deletion of *PMP22* using fluorescence in situ hybridization or multiplex ligation‐dependent probe amplification, and patients with duplication or deletion of *PMP22* were excluded. Clinical information and blood/DNA samples were collected by neurologists or pediatricians and referred to our genetic laboratory at Kagoshima University Hospital. Using the Gentra Puregene Blood kit (QIAGEN), genomic DNA derived from patients and their families was extracted from peripheral blood cells according to the manufacturer's instructions.

### 2.2 Microarray sequencing and whole‐exome sequencing

From April 2007 to April 2012, variant screening was conducted in 417 patients using customized MyGeneChip, CustomSeq, Resequencing Array (Affymetrix, Inc.), targeting 28 disease‐causing or related genes of CMT. We have described the procedure of sequencing and data analysis previously.^10^ However, this methodology could not identify some variants due to the false negative hybridization and a low‐detection efficiency of the DNA microarray in our laboratory.^11^ Thus, we combined whole‐exome sequencing to overcome these issues. Whole‐exome sequencing was performed by HiSeq2000 (Illumina Inc., San Diego). Using the Burrows‐Wheeler Aligner, we aligned the sequences to human genome reference (NCBI37/hg19) and used SAM tools (http://www.htslib.org) for calling the variants. The called variants annotation was performed using CLC Genomic Workbench software program (Qiagen, Hilden, Germany) and an in‐house script. Whole‐exome sequencing was performed as indicated in the previous study.^12^

### 2.3 Targeted resequencing

In May 2012, we introduced the Illumina MiSeq platform (Illumina Inc.), targeting all coding exons and exon–intron junctions of 60 disease‐causing or candidate genes of inherited peripheral neuropathies. We have described this system previously.^13^ We performed variant screening in 437 patients using this sequencing platform, until July 2014. In September 2014, we introduced the Ion Proton System, applying the Ion PI Chip kit v2/v3 BC (Thermo Fisher Scientific, Carlsbad) and began using the Ion AmpliSeq gene panel to target 72 inherited peripheral neuropathy disease‐causing or candidate genes consisting of 1800 amplicons divided into two primers. Variant screening was conducted in 803 patients using this platform, until August 2017.

To analyze the copy number variations of *MPZ*, we screened the 803 patients using CovCopCan software.^14^

### 2.4 Data analysis and variant interpretation

All *MPZ* variants were checked against the Human Gene Mutation Database (https://portal.biobaseinternational.com/hgmd/pro/gene). We then confirmed all variants by checking each variant against the gnomAD browser (https://gnomad.broadinstitute.org) as a global control database and the Human Genetic Variation Database (http://www.hgvd.genome.med.kyoto-u.ac.jp) and the Japanese Multi Omics Reference Panel (https://jmorp.megabank.tohoku.ac.jp/ijgvd/) as Japanese databases to assess whether they were normal variants. We also checked variants against our in‐house database. A series of in silico analyses were executed to predict the pathogenicity of variants using POLYPHEN2 (http://genetics.bwh.harvard.educut/pph2, cut‐off >0.9), SIFT (http://sift.jcvi.org, cut‐off <0.05), PROVEAN (http://provean.jcvi.org/index.php, cut‐off <−2.5), Mutation Taster (http://mutationtaster.org, scores ranging between 0 and 215, variant suspected of pathogenicity is classified as “disease causing” and variant suspected of less pathogenicity is classified as “polymorphism”). We then used Sanger sequencing to validate the suspected variants, and segregation analysis was conducted where possible. Variants were classified according to the American College of Medical Genetics and Genomics and the Association for Molecular Pathology (ACMG/AMP) guidelines published in 2015.^15^

The types and frequency of reported *MPZ* variants in our study were compared with previous studies. We also referred to the reports of *MPZ* variants in Human Genome Mutation Database (HGMD, https://portal.biobaseinternational.com/hgmd/pro/gene). Reports without information regarding the number of patients and related *MPZ* variants were excluded. Patients with *MPZ* variants described in the referenced data were aggregated. To analyze the worldwide mutational distribution of *MPZ* variants, we checked the previous reports described in HGMD. Further, we studied the types of *MPZ* variants and number of patients. Data of *MPZ* variants reported from the same country were also compiled. According to the origin of patients with *MPZ* variants or the country from which the *MPZ* report originated, we classified patients with *MPZ* variants into five regions (Africa, America, Asia, Europe, and Oceania). Our data were classified and aggregated into Asian data. Referenced data in HGMD are described in Supporting information.

### 2.5 Clinical assessment and statistical analysis

Clinical findings and laboratory data of all patients with *MPZ* variants were based on their currently available information. As the large cohort study of *MPZ* variants published in 2015,^8^ the age of onset of patients aged <6, 6–20 and > 20 years was classified as infantile, child and adult onset, respectively.

Patients with median MCV of <38 m/s were classified as demyelinating CMT, and those with median MCV ≥38 m/s were classified as axonal CMT. Patients with deficient electrophysiological findings were designated as unclassified. Serum CK and CSF protein levels were evaluated via blood and CSF tests. We defined elevated CK as serum CK levels >250 IU/L and elevated CSF proteins as CSF protein levels >50 mg/dl. The relationships among age of onset, variant type, CMT type (demyelinating/axonal CMT), CK levels, and CSF protein levels were evaluated. We also evaluated relationship between CSF protein levels and spine MRI findings. Fisher's exact test was used to compare the proportion of patients with adult onset in the elevated and normal CK groups. Proportion of axonal CMT in elevated and normal CK group was also compared using Fisher's exact test. The difference between the proportion of patients with demyelinating and axonal CMT in the elevated and normal CSF protein groups was also evaluated using Fisher's exact test. We considered p‐value of <0.05 as statistical significance. Statistical analysis was performed using R (version 3.6.1 [2019‐07‐05] Copyright 2019). The study protocol was reviewed and approved by the Institutional Review Board of Kagoshima University. Figure S1 shows the schematic diagram of this study.

## 3 RESULTS

### 3.1 Analysis of variants

In 1657 Japanese patients with suspected inherited peripheral neuropathy, we identified 23 known and 17 novel *MPZ* variants in 85 unrelated patients. We confirmed 23 previously reported variants in *MPZ* gene from 64 patients with inherited peripheral neuropathy from different families. The inheritance pattern of the cases was autosomal dominant or sporadic, with 29 (45.3%) patients considered as sporadic cases. The common *MPZ* variants found in our case series were p.Arg98His, p.Thr124Met, p.Asp75Val, p.Arg98Cys, p.Asn35Tyr, and p.Ser78Leu. CNV in the *MPZ* gene have been reported as the cause of inherited peripheral neuropathy,^16^, ^17^ however, none of the patient were confirmed with CNV in *MPZ* gene in the preset study.

Next, we analyzed the differences in worldwide variant distribution. *MPZ* variants observed in more than three regions were considered to be variants distributed worldwide.

**FIGURE 1:**
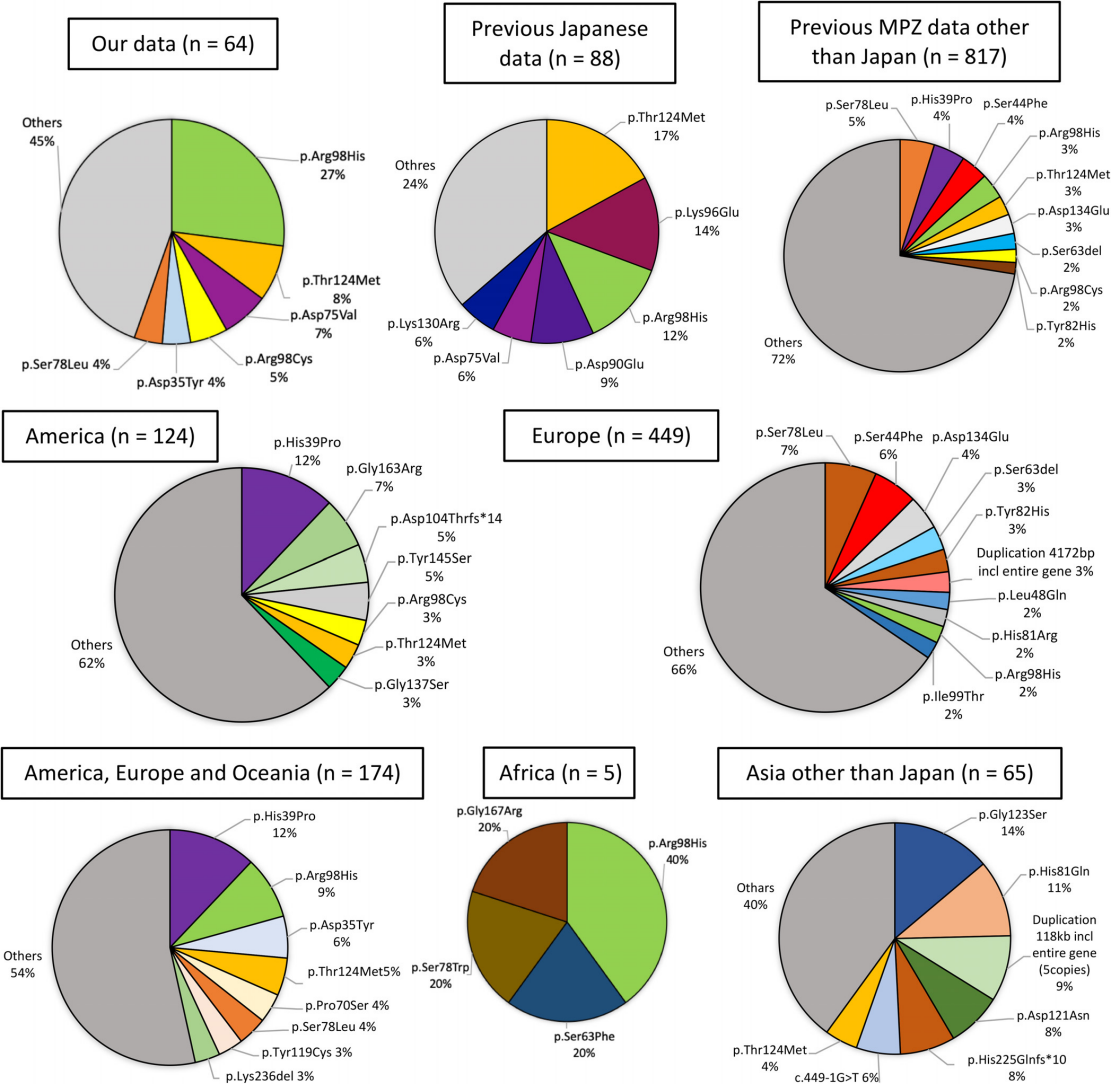
Types and the number of reported *MPZ* variants in our study and previous reports. Types and numbers of reported *MPZ* variants were adopted from HGMD. Cited reports were described in supplemental material

Major *MPZ* variants reported in patients from countries other than Japan were p.Ser78Leu, p.His39Pro, p.Ser44Phe, p.Arg98His, p.Thr124Met, p.Asp134Glu, p.Ser63del, p.Arg98Cys, and p.Tyr82His. Patients with p.His39Pro, p.Ser44Phe, p.Ser63del, p.Tyr82His, and p.Asp134Glu were not detected in our study (Figure 1). We confirmed that 22 variants (p.Arg36Trp, p.Ser44Phe, p.Ser63del, p.Ser63Phe, p.Thr65Ala, p.Ser78Leu, p.Tyr82Cys, p.Arg98Cys, p.Arg98His, p.Gly103Glu, p.Asp104Thrfs*14, p.Ile114Thr, p.Thr124Met, p.Asp128Asn, p.Lys130Arg, p.Ile135Thr, p.Gly137Ser, p.Ser140Thr, p.Gly163Arg, p.Gly167Arg, p.Gln215*, and p.Arg227Ser) were distributed worldwide, whereas six variants (p.Ser44Phe, p.Ser63Phe, p.Thr65Ala, p.Ile135Thr, p.Ser140Thr, and p.Arg227Ser) were not detected in Japan. Moreover, 13 variants (p.Val32Phe, p.Leu48Val, p.Ile62Phe, p.Phe64del, p.Asp75Val, p.Gly93Glu, p.Lys96Glu, p.Asp118_Tyr119insPheTyr, p.Asn131Ser, p.Val146Phe, p.Leu170Arg, p.Ala189Glyfs*47, and p.Arg227Gly) were detected only in Japan (Table 1, Table S1).

**TABLE 1:** Worldwide distribution and number of patients associated with *MPZ* variants. The country names and number of patients are adopted from HGMD. Cited reports are described in supplemental material. The original data for this table is in the supplemental material (Table S1)

Variants	Amino acid change	Europe	America	America, Europe and Oceania	Africa	Asia	Japan
c.94G > T	p.Val32Phe	–	–	–	–	–	1
c.103G > T	p.Asp35Tyr	–	–	10 (Australia)	–	–	3
c.103G > A	p.Asp35Asn	5 (Norway)	–	–	–	–	–
c.106A > T	p.Arg36Trp	1 (UK)	2 (USA)	1 (America, Europe, and Oceania)	–	–	–
c.116A > C	p.His39Pro	–	15 (USA)	21 (America, Europe, and Oceania)	–	–	–
c.131C > T	p.Ser44Phe	26 (Italy)	1 (USA)	4 (America, Europe, and Oceania)	–	–	–
c.143 T > A	p.Leu48Gln	10 (Czech)	–	–	–	–	–
c.143 T > C	p.Leu48Pro	5 (Hungary)	–	–	–	–	–
c.142C > G	p.Leu48Val	–	–	–	–	–	2
c.178G > C	p.Asp60His	9 (Austria)	–	–	–	–	–
c.184A > T	p.Ile62Phe	–	–	–	–	–	4
c.188_190delCCT	p.Ser63del	14 (Netherlands 13, Belgium 1)	–	4 (America, Europe, and Oceania)	–	–	1
c.188C > T	p.Ser63Phe	5 (France)	–	2 (America, Europe, and Oceania)	1 (Algeria)	1 (Taiwan)	–
c.190_192delTTC	p.Phe64del	–	–	–	–	–	4
c.193A > G	p.Thr65Ala	1 (Poland)	1 (USA)	3 (America, Europe, and Oceania)	–	–	–
c.224A > T	p.Asp75Val	–	–	–	–	–	10
c.233C > T	p.Ser78Leu	30 (Belgium 3, Italy 6, France 4, Finland 5, Serbia 6, Spain 1, Switzerland 1, UK 4)	2 (USA)	7 (America, Europe, and Oceania)	–	–	3
c.233C > G	p.Ser78Trp	–	–	–	1 (Nigeria)	–	–
c.242A > G	p.His81Arg	10 (UK)	–	–	–	–	–
c.243C > G	p.His81Gln	–	–	–	–	7 (Korea)	–
c.245A > G	p.Tyr82Cys	1 (Finland)	3 (USA)	2 (America, Europe, and Oceania)	–	–	5
c.244 T > C	p.Tyr82His	13 (Netherlands)	–	–	–	–	‐
c.270C > A	p.Asp90Glu	4 (Cyprus 3, Spain 1)	–	–	–	–	8
c.278G > A	p.Gly93Glu	–	–	–	–	–	3
c.286A > C	p.Lys96Glu	–	–	–	–	–	12
c.292C > T	p.Arg98Cys	6 (Belgium 2, Austria 1, France 1, Italy 1, Spain 1)	4 (USA)	4 (America, Europe, and Oceania)	–	1 (Taiwan)	4
c.293G > A	p.Arg98His	10 (France 3, Switzerland 2, Belgium 1, Italy 1, Russia 1, European countries 2)	1 (USA)	15 (America, Europe, and Oceania)	2 (Algeria)	1 (China)	31
c.293G > C	p.Arg98Pro	7 (France)	–	–	–	–	–
c.296 T > C	p.Ile99Thr	10 (UK)	–	2 (America, Europe, and Oceania)	–	–	–
c.308G > A	p.Gly103Glu	3 (UK)	–	2 (America, Europe, and Oceania)	–	–	1
c.306delA	p.Asp104Thrfs*14	1 (Italy)	6 (USA)	2 (America, Europe, and Oceania)	–	–	–
c341T > C	p.Ile114Thr	1 (USA)	–	2 (America, Europe, and Oceania)	–	–	2
c.355_356insTCTACT	p.Asp118_Tyr119insPheTyr	–	–	–	–	–	1
c.356A > G	p.Tyr119Cys	5 (Germany 3, European countries 2)	‐	6 (America, Europe, and Oceania)	–	–	–
c.361G > A	p.Asp121Asn	–	–	–	–	5 (China)	–
c.367G > A	p.Gly123Ser	–	–	2 (America, Europe, and Oceania)	–	9 (Taiwan)	–
c.371C > T	p.Thr124Met	6 (Italy 5, Germany 1)	4 (USA)	9 (America, Europe, and Oceania)	–	3 (China)	21
c.382G > A	p.Asp128Asn	2 (UK)	1 (USA)	–	–	–	1
c.389A > G	p.Lys130Arg	2 (Belgium)	1 (USA)	2 (America, Europe, and Oceania)	–	1 (China)	7
c.392A > G	p.Asn131Ser	–	–	–	–	–	1
c.400G > A	p.Asp134Asn	6 (Belgium)	–	–	–	–	–
c.402C > A	p.Asp134Glu	20 (Belgium 19, Russia 1)	–	2 (America, Europe, and Oceania)	–	–	–
c.404 T > C	p.Ile135Thr	2 (UK 1, Russia 1)	2 (USA)	5 (America, Europe, and Oceania)	–	–	–
c.409G > A	p.Gly137Ser	1 (UK)	4 (USA)	5 (America, Europe, and Oceania)	–	–	2
c.418 T > A	p.Ser140Thr	1 (European country)	2 (USA)	2 (America, Europe and Oceania)	–	–	–
c.434A > C	p.Tyr145Ser	–	6 (Costa Rica)	3 (America, Europe, and Oceania)	–	–	–
c.436G > T	p.Val146Phe	–	–	–	–	–	1
c.487G > A	p.Gly163Arg	1 (Belgium)	1 (USA)	–	–	–	1
c.499G > A	p.Gly167Arg	2 (UK)	1 (USA)	2 (America, Europe, and Oceania)	1 (Kenya)	–	1
c.509 T > G	p.Leu170Arg	–	–	–	–	–	2
c.560_563dupAGGC	p.Ala189Glyfs*47	–	–	–	–	–	1
c.611A > T	p.Lys204Met	7 (Spain)	–	–	–	–	‐
c.643C > T	p.Gln215*	2 (Italy)	2 (USA)	2 (America, Europe, and Oceania)	–	–	1
c.670G > T	p.Asp224Tyr	9 (Germany 4, Italy 4, Austria 1)	–	–	–	–	–
c.674dupA	p.His225Glnfs*10	–	–	–	–	5 (China)	–
c.679A > G	p.Arg227Gly	–	–	–	–	–	1
c.681A > T	p.Arg227Ser	2 (Serbia)	1 (USA)	2 (America, Europe, and Oceania)	–	–	–
c.699_702delTGAG	p.Ser233Argfs*18	5 (Italy)	–	–	–	1 (Taiwan)	–
c.706_708delAAG	p.Lys236del	–	2 (USA)	6 (America,Europe, and Oceania)	–	–	–
Duplication118 kb inclentire gene(5 copies)	–	–	–	–	–	6 (Taiwan)	–
Duplication4172 bp inclentire gene	–	12 (Norway)	–	–	–	–	–
Patients with other variants		192	62	45	0	15	17

Abbreviations: n, number of patients with inherited peripheral neuropathy having *MPZ* variants.

### 3.2 Analyses of novel *MPZ* variants

We found 17 novel *MPZ* variants from 21 patients and assessed each variant based on ACMG/AMG guidelines. The pedigree trees of 21 patients with 17 novel variants are described in Figure S2. Ten of these (p.Phe19Ser, p.Phe19Ser/p.Asp75Val, p.Ser54Tyr, p.Asp75Gly, p.His81Asp, p.Trp101Arg, p.Ser111Tyr, p.Ile112Val, p.Asn122Asp, and p.Val142Asp) fulfilled three categories of moderate pathogenic evidence. One variant (p.Glu37Lys) fulfilled two categories of moderate pathogenic evidence and two supportive pathogenic evidences. Thus, 11 variants were classified as likely pathogenic and considered as novel pathogenic variants. The six remaining variants (p.Ala5Glyfs*52, p.Val31Leu, c.234 + 1G > A, p.Val102Gly, p.Tyr119dup, and c.646‐3C > G) were classified as uncertain significance or likely benign (Table 2). The mutation sites associated with novel missense variants of likely pathogenic were preserved among mammalians and located in mutational hot spots (Figure S3). p.Phe19Ser, p.Glu37Lys, p.Asp75Gly, p.Ile112Val, p.Asn122Asp, and p.Val142Asp were associated with adult onset, whereas p.Ser54Tyr, p.His81Asp, p.Trp101Arg, and p.Ser111Tyr were associated with child and infantile onset, respectively. p.His81Asp, p.Ser111Tyr, and p.Val142Asp were classified as demyelinating CMT, whereas p.Phe19Ser, p.Glu37Lys, p.Asp75Gly, and p.Ile112Val were classified as axonal CMT (Table 3). We compared the clinical features of 17 novel variants with the reported variants that occurred near or at the same codon. The clinical features of p.Ala5Glyfs*52 and p.Tyr119dup were compared with the reported small insertions. c.234 + 1G > A and c.646‐3C > G were compared with variants occurring at intron. We observed that some variants had similar phenotype as the reported variants, which are occurred near or at the same codon. Patients with p.Glu37Lys, p.Arg36Gly, or p.Arg36Trp had adult onset and they were classified as axonal CMT. Patients with p.Asp75Gly or p.Asp75Val also had the similar phenotype. Although, patients with p.Asn122Asp or p.Asn122Ser were associated with adult onset, the electrophysiological classification varied in each patient. Meanwhile, patients with p.Ser111Tyr, p.Ser111Cys, p.Ser111Phe or p.Ser111Pro had infantile or child onset (Table 3).

**TABLE 2:** Novel *MPZ* variants not previously reported

ID	Nucleotide change	Amino acid change	Control database		In‐silico analysis				ACMG standard and guidelines			Number of patients
			Global database	Japanese and in‐house database	PROVEAN	SIFT	Polyphen 2	Mutation taster	Pathogenicity	Benign impact	Criteria	
7689	c.13dup	p.Ala5Gly fs*52	–	–				Disease causing	PM2, 4		Uncertain significance	1
7484	c.56 T > C	p.Phe19Ser	–	–	−1.39	0.233	0.967	Disease causing	PS4‐moderate, PM1, 2, PP3	BP4	Likely pathogenic (iv)	1
7344	c.56 T > C	p.Phe19Ser	–	–	−1.39	0.233	0.967	Disease causing	PS4‐moderate, PM1, 2, PP3	BP4	Likely pathogenic (iv)	1
	c.224A > T	p.Asp75Val	Reported variant (Misu K et al. 2000)									
391 874 208 166	c.91G > T	p.Val31Leu	–	jMorp ToMMo 4.7KJPN 0.0004	−1.11	0.07	0.328	Disease causing	PS4‐moderate, PM1	BS1, BP4	Likely benign (i)	3
5527	c.109G > A	p.Glu37Lys	–	–	−2.95	0	0.992	Disease causing	PM1, 2, PP1, 3		Likely pathogenic (v)	1
8160	c.161C > A	p.Ser54Tyr	–	–	−5.66	0	1	Disease causing	PM1, 2, 5, 6, PP3		Likely pathogenic (iv)	1
6181	c.224A > G	p.Asp75Gly	–	–	−2.39	0.16	0.987	Disease causing	PM1, 2, 5, PP1, 3	BP4	Likely pathogenic (iv)	1
6621	c.234 + 1 G > A		–	–					PM2, PP1, 4		Uncertain significance	1
ID	Nucleotide change	Amino acid change	Control database		In‐silico analysis				ACMG standard and guidelines			Number of patients
			Global database	Japanese and In‐house database	PROVEAN	SIFT	Polyphen 2	Mutation taster	Pathogenicity	Benign impact	Criteria	
6132 7199	c.241 C > G	p.His81Asp	–	–	−7.04	0.01	0.535	Disease causing	PS4‐moderate, PM1, 2, 5, PP3		Likely pathogenic (iv)	2
4407	c.301 T > C	p.Trp101Arg	–	–	−12.37	0	0.998	Disease causing	PM1, 2, 5, PP1, 3, 4		Likely pathogenic (iv)	1
6125	c.305 T > G	p.Val102Gly	–	–	−2.49	0.02	0.996	Disease causing	PM1, 2, PP3		Uncertain significance	1
7389	c.332C > A	p.Ser111Tyr	–	–	−5.69	0	1	Disease causing	PM1, 2, 5, 6, PP3		Likely pathogenic (iv)	1
5231	c.334A > G	p.Ile112Val	–	–	−0.88	0.02	0.152	Disease causing	PM1, 2, 5, PP3	BP4	Likely pathogenic (iv)	1
5038	c.355_6insACT	p.Tyr119dup	–	–				Polymorphism	PM2, 4, PP1		Uncertain significance	1
4347 5037	c.364A > G	p.Asn122Asp	–	–	−4.66	0.01	0.743	Disease causing	PS4‐moderate, PM1, 2, 5, PP3		Likely pathogenic (iv)	2
7163	c.425 T > A	p.Val142Asp	–	–	−4.81	0	1	Disease causing	PM1, 2, 5, PP3		Likely pathogenic (iv)	1
7393	c.646–3 C > G		2/250518	–					PP1, 4		Uncertain significance	1

*Note*: Likely Pathogenic (iv), Variants fulfill three categories of moderate pathogenic evidence; Likely Pathogenic (v), Variants fulfill two categories of moderate pathogenic evidence and two supportive pathogenic evidences; Likely Benign (i), Variants fulfill one category of strong benign evidence and one category of supporting benign evidence.

**TABLE 3:** Clinical features associated with novel *MPZ* variants detected in this study and reported variants occurring near or at the same site

*MPZ* variants	Age	Onset	Inheritance pattern	Median MCV (m/s)	Demyelinating/axonal CMT	Clinical and laboratory findings	Reference
p.Phe19Ser	56	51	Sp	40.5	A	Elevated CSF protein (52 mg/dl)	This report (ID 7484)
p.Phe19Ser	77	30	AD	31.1	D	Moderate weakness of lower limbs	This report (ID 7344)
p.Asp75Val							
p.Ser20Phe	64	59	AD	Normal	A	Weakness of lower limbs Wasting of lower leg muscles	Finsterer J, et al Eur J Neurol. 2006; 13: 1149–1152
p.Ser20Pro	20	0	–	–	U	–	Milley GM, et al Neuromuscul disord 2018; 28:38–43
p.Val31Leu	16	14	AD	20	D	Mild weakness of all limbs	This report (ID 3918)
p.Val31Leu	73	63	Sp	20.8	D		This report (ID 7420)
p.Val31Leu	58	12	–	29.5	D	Mild weakness of all limbs	This report (ID 8166)
p.Ile30Phe	–	2	AD	–	U	Delayed motor milestones Steppage gait. Pes cavus	Niermeijer JMF, et a Neuromuscul Disort. 2011; 26: 688
p.Ile30Met	39	<3	AD	25	D	Weakness of upper and lower limb muscles Atrophy of all limbs Absent deep tendon reflexes Sensory disturbance Romberg's sign positive	Hayasaka K, et al Hum Mol Genet 1993; 2: 1369
p.Ile30Met	–	early onset	AD	–	U	Distal weakness of the limbs	
p.Ile30Met	–	early onset	AD	–	U	Distal weakness of the limbs	
p.Ile30Met	–	early onset	AD	–	U	Distal weakness of the limbs	
p.Ile30Ser	9	2	–	4	D	Weakness of all limbs Sensory disturbance. Pes cavus	Miltenberger‐Miltenyi G, et al Eur J Hum Genet 2009; 17: 1154–1159
p.Ile30Thr	55	3	AD	–	U	Severe weakness and atrophy of all limbs Romberg's sign positive Claw hands. Pes cavus. Hammer toes	Floroskufi P, et al Muscle Nerve 2007; 35: 667–669
p.Ile30Thr	–	0	AD	–	U	Delayed motor milestonesSevere weakness and atrophy Impaired deep sense. Steppage gait	
p.Val32Phe	26	–	–	15.6	D	–	Yoshihara T, et al Hum Mutat 2000; 16: 177–178
p.Glu37Lys	59	39	AD	55.9	A	Severe weakness of lower limbs	This report (ID 5527)
p.Arg36Gly	77	73	AD	50	A	Weakness of lower limbs Wasting of leg musclesSensory disturbance Romberg's sign. Pes cavus	Dacci P, et al J Peripher Nerv Syst 2012; 17: 422–425
p.Arg36Gly	47	–	AD	–	U	Sensory disturbance	
p.Arg36Trp	47	44	AD	45.6	A	Impaired supefricial and deep sense in feet Pes planus Elevated CSF protein (60 mg/dl)	Burs TM, et al Neuromuscul Disord 2006; 16: 308–310
p.Ser54Tyr	2	0	Sp	–	U	Mild weakness of upper limbs Elegated CSF protein	This report (ID 8160)
p.Ser54Cys	–	–	–	–	D	Classified as CMT1	Hoyer H, et al Biomed Res Int 2014: 13
p.Ser54Pro	26	–	Sp	22	D	Distal weakness Impaired vibratory sensation in lower limbs Pes cavus. Hammer toes	Baissar‐Tadmouri N, et al Hum Mutat 1999; 14: 199
p.Asp75Gly	59	57	Sp	47	A	Mild weakness of lower limbsElevated CK	This report (ID 6181)
p.Asp75Val	74	60	–	52	A	Moderate weakness of upper limbs Severe weakness of lower limbs Sensory disturbance	Misu K, et al J Neurol Neurosurg Psychiatry 2000; 69: 806–811
	66	61	–	44	A	Severe weakness of lower limbs Sensory disturbance. Elevated CK	
	64	61	–	50	A	Mild weakness of lower limbs Sensory diturbance	
p.His81Asp	14	0	AD	11.3	D	Moderate weakness of lower limbs Elevated CSF protein	This report (ID 6132)
p.His81Asp	12	0	AD	9.1	D	Mild weakness of lower limbs	This report (ID 7199)
p.His81Arg	–	–	AD	–	U	–	Sorour E, et al Hum Mutat 1997;9: 74–77
p.His81Arg	28	–	AD	11.1	D	Walking difficulties. Sensory ataxia Foot deformities	Jonathan Beats et al Brain 2011; 134: 2665–2676
p.His81Gln	44	8	AD	N.D	U	Severe muscle atrophy. Ataxia Adie's pupil. Tremor	Choi BO et al Int J Mol Med 2011; 28: 389–396
	40	9	AD	12.2	D	Moderate muscle atrophy Ataxia. Adie's pupil	
	12	5	AD	9.1	D	Mild muscle atrophy Ataxia. Adie's pupil	
	8	2	AD	8.2	D	Mild muscle atrophyAtaxia. Adie's pupil	
	3	2	AD	6.2	D	–	
	13	5	AD	11.9	D	Moderate muscle atrophy Ataxia. Adie's pupil	
	5	4	AD	9.2	D	Mild muscle atrophy. Ataxia	
p.His81Leu	56	48	AD	42	A	Weakness and atrophy of all limbs Sensory disturbance Gait disturbance. Pes cavus	Liu L, et al J Peripher Nerv Syst 2013; 18: 256–260
	63	52	AD	40	A	Weakness and atrophy of all limbs Sensory disturbance Gait disturbance. Pes cavus	
p.Trp101Arg	22	6	AD	–	U	Severe weakness of all limbs Elevated CSF protein	This report (ID 4407)
p.Trp101Cys	32	early onset	AD	10	D	Atrophy of the distal leg muscles Absence of muscle stretch reflexes Pes cavus. Hammertoes. Scoliosis	Latour P, et al Hum Mutat 1995; 6: 50–54
p.Trp101Cys	7	–	AD	–	U	Walking difficulties. Pes cavus	
p.Val102Gly	67	59	AD	40	A	Mild weakness of lower limbs	This report (ID 6125)
p.Gly103Ala	20	–	AD	–	U	–	Sun Y, et al. Sci Rep 2018; 8: 1–9
p.Gly103Arg	–	–	AD	–	U	–	Dohrn MF, et al Journal of Neurochemistry 2017; 143: 507–522
p.Gly103Trp	20	Infancy	AD	–	U	Delayed motor milestones Severe lower limbs atrophy Pes cavus. Gait disturbance	Brozkova D et al Clin Genet 2010; 78: 81–87
	51	Infancy	AD	–	U	Mild lower limbs atrophy Pes cavus. Scoliosis Gait disturbance	
p.Gly103Glu	14	0	AD	7	D	Delayed motor milestones Weakness and atorphy of all limbs Gait disturbance. Genu recuvatum Claw hands and feet Severe kyphoscoliosis	Fabrizi GM, et al Neurology 2001; 57: 101–105
	10	1	AD	slowing NCV	U	Weakness of all limbs Atrophy of distal limbs Sensory disturbance. Pes cavus Waddling gait and steppage gait	
	36	–	AD	–	–	Pes planus	
p.Ser111Tyr	23	0	Sp	3.8	D	–	This report (ID 7389)
p.Ser111Cys	32	<10	AD	14.6	D	Severe weakness in distal lower limbs Muscle wasting in lower limbs Pes cavus	Mandich P, et al Eur J Hum Genet 2009; 17: 1129–1134
p.Ser111Phe	4	0	–	2.9	D	Hypotonia. Weak crying Elevated CSF protein	Sevilla T, et al J Peripher Nerv Syst 2011; 16: 347–352
p.Ser111Pro	–	Infancy	–	–	U	–	Sanmaneechai O, et al Brain 2015; 138: 3180–3192
p.Ile112Val	78	58	Sp	51.3	A	Severe weakness of all limbs Elevated CSF protein (115 mg/dl)	This report (ID 5231)
p.Ile112Thr	–	–	AD	–	U	Severe weakness of all limbs	Sorour E et al Hum Mutat 1998; S1: S242‐247
p.Asn122Asp	68	43	AD	31	D	Elevated CK (297 U/L) and CSF protein (108 mg/dl)	This report (ID 4347)
p.Asn122Asp	47	42	AD	39	A	–	This report (ID 5037)
p.Asn122Ser	53	44	Sp	32	D	Severe scoliosis	Blanquet‐Grossard F, et al Hum Mutat 1996; 8: 185–186
p.Val142Asp	53	43	AD	16	D	Severe weakness of all limbs	This report (ID 7163)
p.Val142Phe	–	Infancy	–	–	U	–	Sanmaneechai O, et al Brain 2015; 138: 3180–3192
c.234 + 1 G > A	51	36	AD	29.5	D	Severe weakness of all limbs	This report (ID 6621)
c.646‐3C > G	44	43	AD	31.1	D	–	This report (ID 7393)
c.235‐2A > C	–	–	–	–	U	–	DiVincenzo C, et al Mol Genet Genomic Med. 2014; 2(6): 522–529
c.449‐9C > T	46	26	–	–	U	Distal weakness Sensory disturbance	Kecharevic‐Markovic M, et al J Peripher Nerv Syst 2009; 14: 125–136
c.449‐1G > A	50	50	–	22	D	Weakness of distal legs Impaired deep sense Romberg's sign	Lancaster E, et al Muscle Nerve 2010; 41: 555–558
c.449‐1G > C	–	–	–	–	U	–	Bort S, et al Hum Genet 1997; 99: 746–754
c.449‐1G > C	52	42	–	–	U	Sensory disturbance Elevated CSF protein (70 mg/dl)	Campagnolo M, et al J Peripher Nerv Syst 2020; 25: 19–26
c.449‐1G > T	–	–	–	–	U	–	Choi BO, et al. Hum Mutat 2004; 24: 185–186
c.448 + 1G > A	–	–	–	–	U	–	DiVincenzo C, et al Mol Genet Genomic Med. 2014; 2(6): 522–529
c.448 + 2 T > G	–	–	–	–	U	–	DiVincenzo C, et al Mol Genet Genomic Med. 2014; 2(6): 522–529
c.584 + 2 T > G	55	20s	AD	–	U	Mild weakness of all limbs Sensory disturbance. Hammer toes	Sabet A, et al Neurology 2006; 67: 1141–1146
	33	29	AD	–	U	Mild weakness of distal arms Sensory disturbance	
	23	0	AD	–	U	Mild weakness of distal lower limbs Sensory disturbance. Pes cavus	
	36	–	–	–	U	–	
c.645 + 1G > T	–	42		–	U	Atrophy of hands Sensory disturbance. Pes cavus	Kleffner I, et al J Neurol 2010; 257: 1864–1868
p.Ala5Glyfs*52	63	48	AD	25.5	D	Severe weakness of upper limbs Elevated CK (447 U/L) and CSF protein (116 mg/dl)	This report (ID 7689)
p.Tyr119dup	49	6	AD	38.7	A	Elevated CSF protein (199 mg/dl)	This report (ID 5038)
c.‐10_‐6 dupTGCCC	39	30	AD	54	A	Mild phenotype	Sivera R, et al Neurology 2013; 81: 1617–1625
p.Tyr88*	–	–	–	–	U	–	DiVincenzo C, et al Mol Genet Genomic Med. 2014; 2(6): 522–529
p.Asp118_Tyr119 insPheTyr	0	0	–	–	U	Clinically severe Diaphragmatic weakness Died at the age of 10 months	Ikegami T, et al Hum Mutat 1998; S1: S103‐105
p.Tyr145Leufs*4	41	40	Sp	38	A	Weakness Muscle wasting in lower legs Pes cavus	Mandich P, et al Eur J Hum Genet 2009; 17: 1129–1134
p.Pro151Alafs*3	34	27	AD	36.8	D	Pes cavus	Piazza S, et al Neuromuscul Disord 2010; 20: 817–819
	–	–	AD	45.2	A	–	
p.Leu184Alafs*51	0	0	AD	4	D	Hypotonia Arthrogryposis of hands and feet	Smit LS, et al Neuromuscul disord 2008; 18: 56–62
	31	0	AD	–	U	Delayed motor milestones Gait disturbance	
p.Ala189Glyfs*47	8	0	Sp	–	U	Delayed motor milestones Weakness of lower limbs Sensory disturbance	Tachi N, et al J Neurol Sci 1998; 156: 167–171
p.Met197Tyrfs*38	35	0	Sp	–	U	Delayed motor milestones Weakness of distal limbs Deformities of limbs and body Sensory disturbance	Zschuntzsch J, et al J Neurol Sci 2009; 281: 113–115
p.Lys207Asnfs*51	–	–	–	–	U	–	Bort S, et al Hum Genet 1997; 99: 746–754
–	26	–	AD	36.9	D	Sensory disturbance	Crehalet H, et al Neurogenetics 2010; 11: 13–19
p.Thr216Asnfs*19	–	–	–	–	U	–	DiVincenzo C, et al Mol Genet Genomic Med. 2014; 2(6): 522–529
p.His225Glnfs*10	–	25	AD	–	U	Weakness and atrophy of distal limbs Sensory disturbance Pes cavus	He J, et al J Peripher Nerv Syst.2018; 23: 216–226

Abbreviations: A, axonal CMT; AD, autosomal dominant; CK, creatine kinase; CSF, cerebrospinal fluid; D, demyelinating CMT; −, Not available or not evoked; NCV, nerve conduction velocity; Sp, Sporadic; U, unclassified type.

A patient had compound heterozygous variant (p.Asp75Val/p.Phe19Ser) with one reported variant (p.Asp75Val) and one novel variant (p.Phe19Ser). This patient had numbness and muscle weakness in legs since his 30s. The brother and nephew of the patient also had difficulty in walking. The nerve conduction velocity of the right median nerve was 31.1 m/sec, which indicated demyelinating CMT. The age of onset in our case series was earlier than in patients with p.Asp75Val in our case series (30 years old vs the average age of 48 years). The daughter and nephew of the patient had one variant (p.Asp75Val). The nephew had weakness, atrophy of all limbs, hyporeflexia of tendon reflexes, pes cavus, and walked with a cane support. The patient's daughter did not have weakness, sensory impairment and decreased tendon reflexes, and denied to undergo electrophysiological examination. Presently, the age of the daughter is 50 years old and she does not have symptoms associated with neuropathy. The nephew's age of onset (56 years) was older than that of the patient (56 years old vs 30 years old; Figure S4).

### 3.3 Clinical and laboratory findings

**FIGURE 2:**
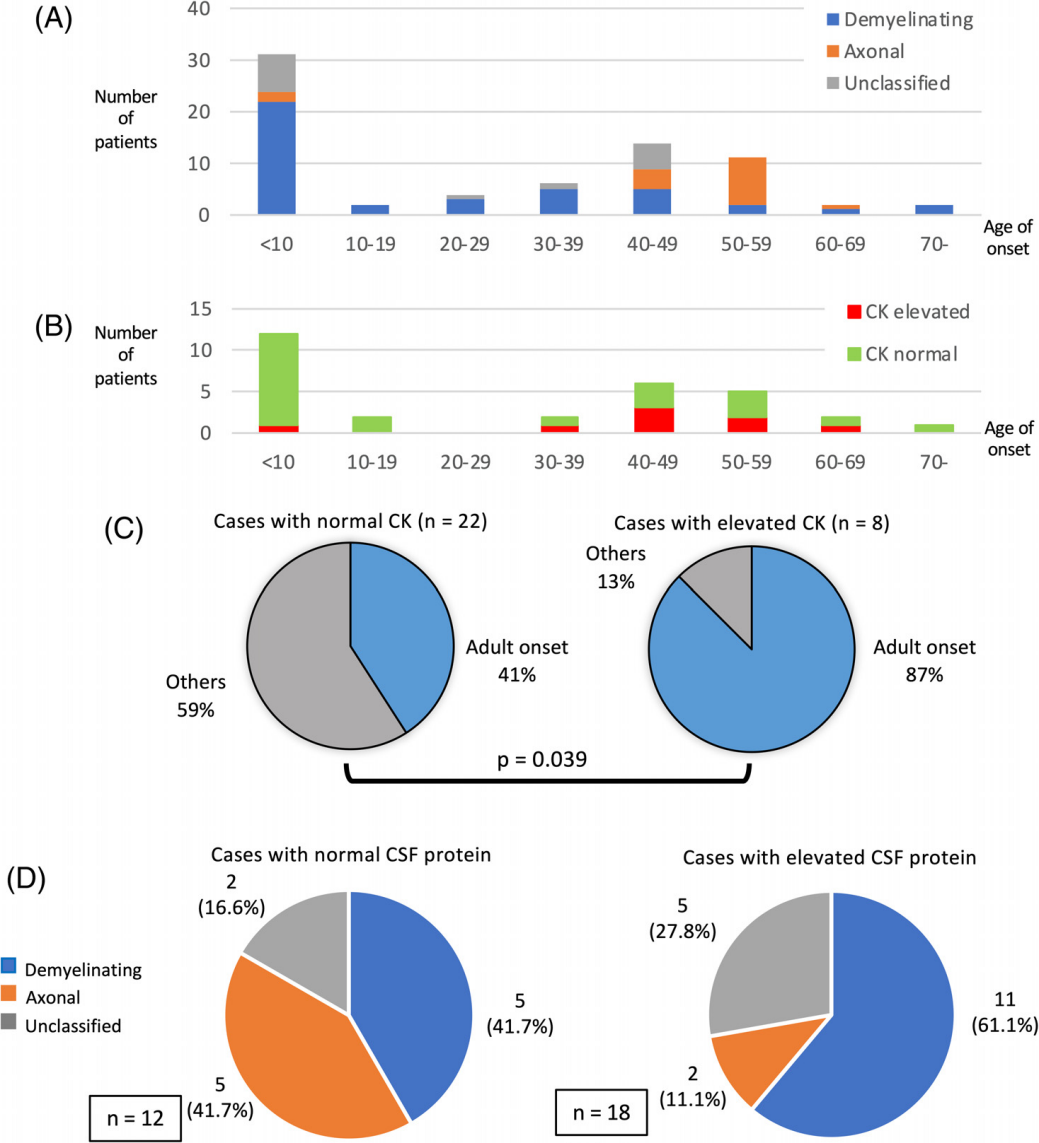
(A) Age of onset and number of patients with *MPZ* variants. (B) Age of onset and number of patients with elevated and normal CK. (C) Proportion of patients with adult onset in the elevated and normal CK groups. (D) The proportion of demyelinating, axonal, and unclassified CMT among the elevated and normal CSF protein groups

We assessed 77 patients with inherited peripheral neuropathy comprising 64 with reported *MPZ* variants and 13 with novel pathogenic *MPZ* variants (Table S2). The onset age of these patients indicated a bimodal distribution (Figure 2(A)). Prominent clustering in the first decade and slight clustering between the third and fifth decade were evident, in line with large genetic profiles of Japanese CMT patients.^9^

Cranial nerve involvement was confirmed in 20 patients (Table S3). Dysarthria was detected in seven patients, while dysphagia was confirmed in four patients. Hearing loss was also detected in four patients (Table 4). The most common *MPZ* variant in patients presenting with cranial nerve involvement was p.Arg98His. Furthermore, patients with p.Arg98Cys, p.Asp35Tyr, p.Leu48Val, p.Asp61Asn, p.Asp75Val, p.Phe19Ser/p.Asp75Val, p.Gly103Glu, p.Ile112Val, p.Ile114Thr, p.Thr124Met, p.Asp128Asn, p.Lys130Arg, and p.Leu170Arg showed symptoms associated with cranial nerve dysfunction. The clinical information of patient with p.Thr124Met has been described elsewhere.^18^

**TABLE 4:** Cranial nerve involvement and associated *MPZ* variants

Cranial nerve involvement	Variants	Number of patients
Dysarthria	p.Leu48Val, p.Asp75Val, p.Phe19Ser/p.Asp75Val, p.Arg98Cys, p.Arg98His (2), p.Asp128Asn	7
Dysphagia	p.Asp75Val, p.Arg98His (2), p.Asp128Asn	4
Hearing loss	p.Leu48Val, p.Gly103Glu, p.Lys130Arg, p.Leu170Arg	4
Anisocoria	p.Leu48Val, p.Phe19Ser/p.Asp75Val, p.Thr124Met	3
Weakness of facial muscle	p.Asp61Asn, p.Arg98His (2)	3
Deviation of tongue protrusion	p.Arg98His (2), p.Leu170Arg	3
Nystagmus	p.Phe19Ser/p.Asp75Val, p.Arg98His	2
Strabismus	p.Lys130Arg, p.Leu170Arg	2
Atrophy of facial muscle	p.Asp35Tyr, p.Arg98His	2
Atrophy of tongue	p.Asp128Asn, p.Leu170Arg	2
Adie's pupil	p.Thr124Met	1
Ptosis	p.Ile112Val	1
Sluggish light reflex	p.Leu48Val	1
Trigeminal neuralgia	p.Ile114Thr	1
Facial nerve paralysis	p.Leu170Arg	1
Tinnitus	p.Arg98His	1
Atrophy of trapezius and sternocleidomastoid	p.Asp61Asn	1
Involuntary movement of tongue	p.Arg98Cys	1
Tongue fasciculation	p.Asp128Asn	1

Abbreviations: p.Arg98His (2), Two patients with *MPZ* p.Arg98His variant.

We analyzed serum CK levels in 30 patients. Of them, eight (26.7%) showed elevated CK levels, with the levels being <1000 U/L in most cases. Most of the patients with elevated CK levels had neuropathic symptoms in their middle age (Figure 2(B)). The proportion of patients with adult onset was greater in the elevated CK group than in the normal CK group (p = 0.039) (Figure 2(C)). However, patients with elevated CK were not statistically associated with axonal CMT (p = 0.57) (Table S4).

We analyzed CSF protein levels in 30 patients, 18 (60%) of whom had elevated levels. Among patients with elevated CSF protein levels, 11 (61.1%) patients were classified as demyelinating and 2 (11.1%) were classified as axonal CMT. There was no significant difference between the proportion of patients with demyelinating and axonal CMT in the elevated and normal CSF protein groups (p = 0.168) (Figure 2(D)). Eight patients with elevated CSF protein levels had spine MRIs, and four (50%) of these had spinal diseases such as spinal canal stenosis or cervical spondylosis. Two (25%) patients had enlarged nerve root or cauda equina (Table S5).

## 4 DISCUSSION

We investigated 85 patients with inherited peripheral neuropathy associated with *MPZ* variants in Japan. In this study, we focused on the distribution of *MPZ* variants in the world to compare Japanese patients with known *MPZ* variants included in our case series. Interestingly, there were differences in the types of *MPZ* variants between Japan and other countries. In the present study, we confirmed 13 variants, which have been reported only in Japan. However, one of the 13 variants (p.Leu48Val) was reported from Russia.^19^ Therefore, patients with 12 variants were considered to be concentrated in Japan. Three of the 12 variants (p.Asp75Val, p.Gly93Glu, and p.Leu170Arg) were also detected in our study. Patients with p.Asp75Val were frequently observed and described in a study of axonal CMT in Japan.^20^, ^21^ p.Gly93Glu was detected in a Japanese CMT1B family with low‐nerve conduction velocities.^22^ p.Leu170Arg was described in large study analyzing 161 CMT patients for *PMP22*, *GJB1*, and *MPZ*.^20^ Although not explored in the present study, there may be various factors including founder effect and/or difference of ethnicity.

Herein, we detected 11 confirmed novel variants that are likely to induce a pathogenic phenotype. Remarkably, p.Glu37Lys, p.Asp75Gly, and p.Ser111Tyr were associated with a similar phenotype as the reported variants that occurred at and near the same codon. Therefore, the confirmed novel variants likely induced a pathogenic phenotype, especially in these missense variants. Furthermore, in one of the patients with a novel pathogenic variant, a compound heterozygous variant of p.Asp75Val and p.Phe19Ser was confirmed. The patients with p.Asp75Val are often classified as axonal CMT with late onset of neuropathic symptoms.^21^ Compound heterozygous variants have been previously observed in some genes associated with autosomal dominant CMT (*PMP22*, *MFN2*, *GDAP1*, etc.) and have contributed to unusual phenotype.^23^, ^24^ The cumulative effect of two mild variants was hypothesized in a CMT family with simultaneous *MFN2* and *GDAP1* variants.^24^ To the best of our knowledge, there have been a few compound heterozygous variants of *MPZ*.^25^, ^26^, ^27^ Regarding the compound heterozygous variant in our study, this patient was classified as demyelinating CMT. In addition, he had younger age of onset than those with p.Asp75Val and showed demyelinating neuropathy on nerve conduction studies. The clinical findings of compound heterozygous variant of p.Asp75Val and p.Phe19Ser seem to be different from those of p.Asp75Val. Thus, the patient with p.Asp75Val and p.Phe19Ser had atypical phenotype compared to patient with p.Asp75Val. However, the pedigree tree for this compound heterozygous variant indicated the possibility that p.Asp75Val and p.Phe19Ser was located in each allele. Thus, the pathogenicity of p.Phe19Ser in this patient was unclear and p.Phe19Ser may not contribute to atypical phenotype of the patient. Accumulation of the clinical information about the same variant in more patients and functional studies to prove the pathogenicity of p.Phe19Ser must be performed. Variants of two genes related to the protein that are synergetic in the same pathway can cause overlapping disease phenotype, which may contribute to the atypical phenotype of mendelian disorder.^28^ Thus, the factor such as other genes related to the protein interacting MPZ protein should be considered if the pathogenicity of p.Phe19Ser is denied.

Cranial nerve involvement is rarely seen in CMT.^29^ However, hearing loss should be carefully discussed considering the involvement of the cranial nerve in *MPZ* variants. The frequency of hearing loss in 66 CMT patients with *MPZ* variants was reported as 3.33%, suggesting that the frequency of hearing loss is the same as that in the normal population. Thus, hearing loss may not be associated with *MPZ* variants.^30^ However, in addition to hearing loss, pupillary abnormalities, trigeminal neuralgia, hemifacial spasm have been observed in CMT patients with *MPZ* variants.^31^, ^32^, ^33^ Further, pupillary abnormalities such as Adie's pupil have been described in association with *MPZ* variants and autonomic nervous dysfunction.^18^, ^34^, ^35^, ^36^ Moreover, the number of patients with cranial nerve involvement except hearing loss detected in the present study was 19. Eleven patients had more than two symptoms related to cranial nerve involvement. These findings suggest that various cranial nerve involvement can be observed in some CMT patients with *MPZ* variants. Therefore, these cranial nerve symptoms may provide clues for examining the *MPZ* variants.

In the present study, we also focused on serum CK and CSF protein levels. Serum CK level elevation has also been detected among demyelinating and axonal CMT patients with *MPZ* variants.^21^, ^37^, ^38^ In our case series, patients with elevated CK levels were more likely to have adult onset than those with normal CK levels. This result is in line with previous studies.^21^ Samaneechai et al. have shown that degeneration of myelinated axons causes peripheral neuropathy in adult onset (aged >20 years) patients.^8^ Moreover, it has been suggested that impaired muscle membrane integrity caused by denervation deriving from impaired axons is involved in CK level elevation.^37^ Therefore, degeneration of myelinated axons and associated denervation may have contributed to CK level elevation.

Elevated CSF protein levels have previously been described in CMT with *MPZ* variants.^21^, ^38^, ^39^, ^40^ Various factors have been considered for elevated CSF protein levels. Half of patients with elevated CSF protein levels had spinal diseases such as spinal canal stenosis and cervical spondylosis. It is unclear whether these spinal diseases are associated with *MPZ* variants. However, these spinal diseases can interrupt CSF flow and increase CSF protein levels.^41^ In the present study, not only patients with demyelinating CMT but also those with axonal CMT had elevated CSF protein levels. In our case series, one axonal CMT patient with an elevated CSF protein had slight enlargement of cauda equina. This finding was described in Italian patient with *MPZ* p.Gly167Arg variant.^42^ It has been reported that the leakage of blood protein caused by the partial impairment of CSF circulation and blood‐nerve barrier injury at enlarged nerve root site can contribute to elevated CSF protein levels.^43^, ^44^, ^45^ Thus, enlargement of cauda equina may be associated with CSF circulation and CSF protein elevation in this case. In contrast, conditions such as spinal diseases, enlarged nerve root or cauda equina were not observed in other axonal CMT patient with an elevated CSF protein. An elevated CSF protein level has been reported in axonal CMT patients with *MPZ* p.Thr124Met variants.^21^ Thus, some *MPZ* variants may be associated with an elevated CSF protein level even in axonal CMT patients. Axonal CMT patients with an elevated CSF protein in this study had novel variants (p.Phe19Ser and p.Ile112Val). These *MPZ* variants may be associated with elevated CSF protein, while the pathogenicity of these variants and association with elevated CSF protein should be analyzed.

There are several points to consider in this study. First, we analyzed the patients with novel variants and assessed their pathogenicity in accordance with the ACMG guidelines. Although the exact pathogenicity of novel variants should be assessed by functional studies, we were unable to perform functional studies for novel *MPZ* variants. Also, we were unable to perform the clinical assessment for severity such as CMT neuropathy score. Further, we were able to analyze serum CK, CSF protein levels and MRI findings only in limited patients. Due to the design of this study, these data were insufficient in this study. These points will be addressed in future studies.

## CONFLICT OF INTEREST

The authors declare no financial or other conflicts of interest.

## ETHICAL STATEMENT

The study protocol was reviewed and approved by the Institutional Review Board of Kagoshima University. All patients and family members provided written informed consent to participate in the study.

### PEER REVIEW

The peer review history for this article is available at https://publons.com/publon/10.1111/cge.13881.

## Supporting information


**Figure S1** Schematic diagram of this study. ACMG/AMP, American College of Medical Genetics and Genomics and the Association for Molecular Pathology. CK, creatine kinase; CSF, cerebrospinal fluid
**Figure S2** Pedigree trees of cases with 17 rare *MPZ* variants. Circles indicates females. Squares indicates males. Gray circles and squares indicate affected family members. Arrows indicates probands
**Figure S3** Sequence alignment for amino acids reveals the novel missense variant sites and mutational hot spots. Variants with red characters indicate novel variants identified in this study. Variants with black characters indicate reported *MPZ* variants
**Figure S4** Pedigree trees of a patient with a novel compound heterozygous *MPZ* variant and age of symptom onset of this family and patients with p.Asp75Val in our study
**Table S1** Worldwide distribution and number of patients associated with *MPZ* variants. Table S1 is summarized to create Table 1
**Table S2** Clinical data of 77 CMT patients with *MPZ* variants in this study. A, axonal CMT; AD, autosomal dominant; CK, creatine kinase; CSF, cerebrospinal fluid; D, demyelinating CMT; Lower limb strength, Lower limb strength scores in CMT neuropathy scores; Sp, Sporadic; Upper limb strength, Upper limb strength scores in CMT neuropathy scores; U, unclassified type; −, Not available or not evoked
**Table S3** Patients with cranial nerve involvement
**Table S4** Numbers of patients with axonal type in elevated and normal CK groups
**Table S5** MRI findings of patients with elevated CSF protein. ‐, not available

## Data Availability

All data generated or analysed during this study are included in this published article and its supplementary information files.
