# Supplementary material for: Genetic spectrum of Charcot–Marie–Tooth disease associated with myelin protein zero gene variants in Japan
Source: Clin Genet. 2020 Nov 27;99(3):359–75. doi: 10.1111/cge.13881 (PMC7898366; doi:10.1111/cge.13881)
Supplement: Supplementary file 1 — Figure S1 Schematic diagram of this study. ACMG/AMP, American College of Medical Genetics and Genomics and the Association for Molecular Pathology. CK, creatine kinase; CSF, cerebrospinal fluid Figure S2 Pedigree trees of cases with 17 rare MPZ variants. Circles indicates females. Squares indicates males. Gray circles and squares indicate affected family members. Arrows indicates probands Figure S3 Sequence alignment for amino acids reveals the novel missense variant sites and mutational hot spots. Variants with red characters indicate novel variants identified in this study. Variants with black characters indicate reported MPZ variants Figure S4 Pedigree trees of a patient with a novel compound heterozygous MPZ variant and age of symptom onset of this family and patients with p.Asp75Val in our study Table S1 Worldwide distribution and number of patients associated with MPZ variants. Table S1 is summarized to create Table 1 Table S2 Clinical data of 77 CMT patients with MPZ variants in this study. A, axonal CMT; AD, autosomal dominant; CK, creatine kinase; CSF, cerebrospinal fluid; D, demyelinating CMT; Lower limb strength, Lower limb strength scores in CMT neuropathy scores; Sp, Sporadic; Upper limb strength, Upper limb strength scores in CMT neuropathy scores; U, unclassified type; −, Not available or not evoked Table S3 Patients with cranial nerve involvement Table S4 Numbers of patients with axonal type in elevated and normal CK groups Table S5 MRI findings of patients with elevated CSF protein. ‐, not available [file CGE-99-359-s001.docx]

[Supplementary material]

**Figure S1.** Schematic diagram of this study. Abbreviations : ACMG/AMP, American College of Medical Genetics and Genomics and the Association for Molecular Pathology ; CK, creatine kinase ; CSF, cerebrospinal fluid

Continued below

Continued below

**Figure S2.** Pedigree trees of cases with 17 rare *MPZ* variants. Circles indicates females. Squares indicates males. Gray circles and squares indicate affected family members. Arrows indicates probands.

**Figure S3.** Sequence alignment for amino acids reveals the novel missense variant sites and mutational hot spots. Variants with red characters indicate novel variants identified in this study. Variants with black characters indicate reported *MPZ* variants.

**Figure S4.** Pedigree trees of a patient with a novel compound heterozygous *MPZ* variant and age of symptom onset of this family and patients with p.Asp75Val in our study.

| Variants | Amino acid change | Europe | America | America, Europe and Oceania | Africa | Asia | Japan |
| --- | --- | --- | --- | --- | --- | --- | --- |
| c.1A>G | p.Met1Val | - | 1 (USA) | - | - | - | - |
| c.59C>T | p.Ser20Phe | 1 (Austria) | - | - | - | - | - |
| c.58T>C | p.Ser20Pro | 4 (Hungary) | - | - | - | - | - |
| c.89T>G | p.Ile30Ser | 1 (Austria) | - | - | - | - | - |
| c.90C>G | p.Ile30Met | 1  (European  country) | - | - | - | 1 (China) | 4 |
| c.89T>C | p.Ile30Thr | 2 (Greece) | - | - | - | - | - |
| c.88A>T | p.Ile30Phe | 1 (Netherland) | - | - | - | - | - |
| c.88_89 delA Tins11 | p.Ile30delins GlyValTyrThr | 1 (Turkey) | - | - | - | - | - |
| c.94G>T | p.Val32Phe | - | - | - | - | - | 1 |
| c.98A>T | p.Tyr33Phe | 1 (Austria) | - | - | - | - | - |
| c.101C>A | p.Thr34Asn | - | - | - | - | 1 (China) | - |
| c.101C>T | p.Thr34Ile | 1 (Belgium) | - | - | - | - | 1 |
| c.103G>T | p.Asp35Tyr | - | - | 10 (Australia) | - | - | 3 |
| c.103G>A | p.Asp35Asn | 5 (Norway) | - | - | - | - | - |
| c.106A>G | p.Arg36Gly | 2 (Italy) | - | - | - | - | - |
| c.106A>T | p.Arg36Trp | 1 (UK) | 2 (USA) | 1 (America, Europe and Oceania) | - | - | - |
| c.116A>C | p.His39Pro | - | 15  (USA) | 21 (America, Europe and Oceania) | - | - | - |
| c.123_125  delTGT/ c.661G>A | p.Val42del/ p.Ala221Thr | 1 (Turkey) | - | - | - | - | - |
| c.129_136  del  CTCCCGGG | p.Ser44Aspfs*10 | 1 (UK) | 1 (USA) | - | - | - | - |
| c.130_137  del  TCCCGGGT | p.Ser44Aspfs*10 | 2 (UK) | - | - | - | - | - |
| c.131C>T | p.Ser44Phe | 26 (Italy) | 1 (USA) | 4 (America, Europe and Oceania) | - | - | - |
| c,136delG | p.Val46* | - | - | 2 (America, Europe and Oceania) | - | - | - |
| c.143T>A | p.Leu48Gln | 10 (Czech) | - | - | - | - | - |
| c.143T>C | p.Leu48Pro | 5 (Hungary) | - | - | - | - | - |
| c.142C>G | p.Leu48Val | - | - | - | - | - | 2 |
| c.148T>G | p.Cys50Gly | - | - | 1 (Austria) | - | - | 1 |
| c.149_151 delGCT | p.Cys50del | - | 1 (USA) | - | - | - | - |
| c.152C>T | p.Ser51Phe | 3  (Italy 1,  Switzerland 2) | - | - | - | - | - |
| c.152C>G | p.Ser51Cys | 1 (UK) | - | - | - | - | - |
| c.156C>G | p.Phe52Leu | - | - | 1 (America, Europe and Oceania) | - | - | - |
| c.154T>G | p.Phe52Val | - | - | - | - | 1 (Korea) | - |
| c.158G>A | pTrp53* | 1 (Denmark) | - | - | - | - | - |
| c.160T>C | p.Ser54Pro | 2  (France, 1  Turkey 1) | - | - | - | - | - |
| c.161C>G | p.Ser54Cys | 1 (Norway) | - | - | - | - | - |
| c.164G>T | p.Ser55Ile | 3  (2 Germany,  UK 1) | - | - | - | - | - |
| c.166G>A | p.Glu56Lys | 3 (Poland) | - | - | - | - | - |
| c.173T>A | p.Val58Asp | - | - | - | - | 1 (Taiwan) | - |
| c.172G>T | p.Val58Phe | 1 (UK) | - | - | - | - | - |
| c.178G>C | p.Asp60His | 9 (Austria) | - | - | - | - | - |
| c.182A>G | p.Asp61Gly | 4 (Germany) | - | - | - | - | - |
| c.181G>A | p.Asp61Asn | 2 (Italy) | - | - | - | - | 3 |
| c.186C>G | p.Ile62Met | 1 (Austria) | - | - | - | - | - |
| c.184A>T | p.Ile62Phe | - | - | - | - | - | 4 |
| c.188_190 delCCT | p.Ser63del | 14  (Netherlands 13,  Belgium 1) | - | 4 (America, Europe and Oceania) | - | - | 1 |
| c.188C>G | p.Ser63Cys | - | - | 1 (Australia) | - | - | - |
| c.188C>T | p.Ser63Phe | 5 (France) | - | 2 (America, Europe and Oceania) | 1 (Algeria) | 1 (Taiwan) | - |
| c.190_192 delTTC | p.Phe64del | - | - | - | - | - | 4 |
| c.193A>G | p.Thr65Ala | 1 (Poland) | 1 (USA) | 3 (America, Europe and Oceania) | - | - | - |
| c.194C>A | p.Thr65Asn | 1 (Czech) | - | - | - | - | - |
| c.194C>T | p.Thr65Ile | - | - | - | - | 2 (Taiwan 1, China 1) | 2 |
| c.197G>A | p.Trp66* | - | 1 (USA) | - | - | - | - |
| c.199C>T | p.Arg67Cys | 1 (UK) | - | - | - | - | - |
| c.199C>T  (+ PMP22  duplication) | p.Arg67Cys  (+PMP22  duplication) | 4 (UK) | - | - | - | - | - |
| c.200G>C  (+ SCN4A  c.2111C>T) | p.Arg67Pro  (+ SCN4A  p.Thr704Met) | - | 2 (USA) | - | - | - | - |
| c.204C>A | p.Tyr68* | 1  (European  country) | 1 (USA) | - | - | - | - |
| c.203A>G | p.Tyr68Cys | 2 (UK) | - | - | - | - | 3 |
| c.205C>T | p.Gln69* | - | - | - | - | 2 (India) | - |
| c.209C>T | p.Pro70Leu | 3 (Cyprus) | - | - | - | - | - |
| c.208C>T | p.Pro70Ser | 3 (Italy) | - | 7 (America, Europe and Oceania) | - | - | - |
| c.211G>T | p.Glu71* | 2 (France) | - | - | - | - | - |
| c.224A>T | p.Asp75Val | - | - | - | - | - | 10 |
| c.233C>T | p.Ser78Leu | 30  (Belgium 3,  Italy 6,  France 4,  Finland 5,  Serbia 6,  Spain 1,  Switzerland 1,  UK 4) | 2 (USA) | 7 (America, Europe and Oceania) | - | - | 3 |
| c.233C>T  (+ GBA  c.1448T>C) | p.Ser78Leu  (+ GBA  p.Leu444Pro) | - | 2 (USA) | - | - | - | - |
| c.233C>G | p.Ser78Trp | - | - | - | 1 (Nigeria) | - | - |
| c.241C>T /c.337G>T | p.His81Tyr /p.Val113Phe | 1  (Netherlands) | - | - | - | - | - |
| c.242A>G | p.His81Arg | 10 (UK) | - | - | - | - | - |
| c.243C>G | p.His81Gln | - | - | - | - | 7 (Korea) | - |
| c.242A>T | p.His81Leu | - | - | - | - | 2 (China) | - |
| c.245A>G | p.Tyr82Cys | 1 (Finland) | 3 (USA) | 2 (America, Europe and Oceania) | - | - | 5 |
| c.245A>C | p.Tyr82Ser | 1 (Italy) | - | - | - | - | - |
| c.244T>C | p.Tyr82His | 13  (Netherlands) | - | - | - | - | - |
| c.254G>C | p.Gly85Ala | 1  (European  country) | - | - | - | - | - |
| c.253G>A | p.Gly85Arg | 3 (Hungary) | - | - | - | - | - |
| c.256C>T | p.Gln86* | - | - | 1 (USA) | - | - | - |
| c.262T>C | p.Tyr88His | - | - | - | - | 1 (Korea) | 1 |
| c.263_264 insACCCTA | p.Tyr88* | - | - | 1 (USA) | - | - | - |
| c.258_265 delACCCTACA insCCTCT | p.Gln86_Ile89 delins HisLeuPhe | 2 (Finland) | - | - | - | - | - |
| c.266T>A, c.274G>A, c.486C>G | p.Ile89Asn, p.Val92Met, p.Ile162Met | - | 1 (USA) | - | - | - | - |
| c.270C>A | p.Asp90Glu | 4  (Cyprus 3,  Spain 1) | - | - | - | - | 8 |
| c.268G>C | p.Asp90His | - | - | 2 (America, Europe and Oceania) | - | - | - |
| c.278G>A | p.Gly93Glu | - | - | - | - | - | 3 |
| c.284T>G | p.Phe95Cys | 1 (Hungary) | - | - | - | - | - |
| c.286A>C | p.Lys96Glu | - | - | - | - | - | 12 |
| c.286A>C  (+ DYNC1H1  c.12804C>T) | p.Lys96Glu  (+ DYNC1H1  p.Phe4268Phe) | 1 (Denmark) | - | - | - | - | - |
| c.290A>T | p.Glu97Val | 3 (Czech) | - | - | - | - | - |
| c.290_293 delAGCG | p.Glu97Alafs*5 | 4 (Czech) | - | - | - | - | - |
| c.292C>T | p.Arg98Cys | 6  (Belgium 2,  Austria 1,  France 1,  Italy 1,  Spain 1) | 4 (USA) | 4 (America, Europe and Oceania) | - | 1 (Taiwan) | 4 |
| c.293G>A | p.Arg98His | 10  (France 3,  Switzerland 2,  Belgium 1,  Italy 1,  Russia 1,  European  countries 2) | 1 (USA) | 15 (America, Europe and Oceania) | 2 (Algeria) | 1 (China) | 31 |
| c.293G>T  (+ MFN2  c.2146G>A) | p.Arg98Leu  (+ MFN2  p.Ala716Thr) | - | 1 (USA) | - | - | - | - |
| c.293G>C | p.Arg98Pro | 7 (France) | - | - | - | - | - |
| c.292C>A | p.Arg98Ser | - | 2 (USA) | - | - | - | - |
| c.296T>C | p.Ile99Thr | 10 (UK) | - | 2 (America, Europe and Oceania) | - | - | - |
| c.303G>C | p.Trp101Cys | 3 (France) | - | - | - | - | - |
| c.298C>T | p.Gln100* | 1 (Austria) | - | - | - | - | - |
| c.307G>T | p.Gly103Trp | 2 (Czech) | - | - | - | - | - |
| c.308G>C | p.Gly103Ala | 1 (Denmark) | - | - | - | - | - |
| c.307G>A | p.Gly103Arg | 1  (European  country) | - | - | - | - | - |
| c.308G>A | p.Gly103Glu | 3 (UK) | - | 2 (America, Europe and Oceania) | - | - | 1 |
| c.306delA | p.Asp104Thrfs*14 | 1 (Italy) | 6 (USA) | 2 (America, Europe and Oceania) | - | - | - |
| c.313C>A | p.Pro105Thr | 2  (Poland 1,  European  country 1) | - | - | - | - | - |
| c.313C>T | p.Pro105Leu | - | 3 (USA) | - | - | - | - |
| c.316C>T | p.Arg106Cys | 3 (Finland) | - | - | - | - | - |
| c.325G>A | p.Asp109Asn | 1 (France) | - | - | - | - | - |
| c.327T>A | p.Asp109Glu | 5 (Italy) | - | - | - | - | - |
| c.329G>A | p.Gl110Asp | 1 (Germany) | - | 3 (America, Europe and Oceania) | - | - | - |
| c.332C>G | p.Ser111Cys | 3 (Italy) | - | 3 (America, Europe and Oceania) | - | - | - |
| c.332C>T | p.Ser111Phe | 1 (Spain) | - | - | - | - | - |
| c.331T>C | p.Ser111Pro | - | - | 3 (America, Europe and Oceania) | - | - | - |
| c.335T>C | p.Ile112Thr | 2 (UK) | - | 2 (America, Europe and Oceania) | - | - | - |
| c.337G>T | p.Val113Phe | - | - | - | - | - | 1 |
| c341T>C | p.Ile114Thr | 1 (USA) | - | 2 (America, Europe and Oceania) | - | - | 2 |
| c.341delT | p.Ile114Asnfs*4 | - | - | 4 (America, Europe and Oceania) | - | - | - |
| c.346A>C | p.Asn116His | - | 1 (USA) | - | - | - | - |
| c.347A>G | p.Asn116Ser | 1 (Germany) | - | - | - | - | - |
| c.352G>A | p.Asp118Asn | - | - | - | - | 2 (Korea) | - |
| c.355_356 insTCTACT | p.Asp118_Tyr119 insPheTyr | - | - | - | - | - | 1 |
| c.356A>G | p.Tyr119Cys | 5  (Germany 3,  European  countries 2) | - | 6 (America, Europe and Oceania) | - | - | - |
| c.361G>A | p.Asp121Asn | - | - | - | - | 5 (China) | - |
| c.365A>G | p.Asn122Ser | 2 (France) | - | - | - | - | - |
| c.368G>A | p.Gly123Asp | 1 (Norway) | - | - | - | - | - |
| c.367G>T | p.Gly123Cys | - | 3 (USA) | - | - | - | - |
| c.367G>A | p.Gly123Ser | - | - | 2 (America, Europe and Oceania) | - | 9 (Taiwan) | - |
| c.368G>T | p.Gly123Val | 2 (Norway) | - | - | - | - | - |
| c.368_382del15 | p.Gly123_Cys127del | - | 1 (USA) | - | - | - | - |
| c.370A>C | p.Thr124Pro | 1 (Hungary) | - | - | - | - | - |
| c.371C>T | p.Thr124Met | 6  (Italy 5,  Germany 1) | 4 (USA) | 9 (America, Europe and Oceania) | - | 3 (China) | 21 |
| c.370A>G | p.Thr124Ala | 1 (Italy) | - | - | - | - | - |
| c.371C>A | p.Thr124Lys | 1 (Poland) | - | - | - | - | - |
| c.380G>C | p.Cys127Ser | - | 1 (USA) | - | - | - | - |
| c.380G>A | p.Cys127Tyr | 1 (Italy) | - | - | - | - | - |
| c.382G>A | p.Asp128Asn | 2 (UK) | 1 (USA) | - | - | - | - |
| c.372_377 delGTTCAC | p.Phe125_Thr126del | 1 (Italy) | - | - | - | - | - |
| c.382G>A | p.Asp128Asn | 2 (UK) | 1 (USA) | - | - | - | 1 |
| c.383A>G | p.Asp128Gly | 1 (UK) | - | - | - | - | - |
| c.389A>G | p.Lys130Arg | 2 (Belgium) | 1 (USA) | 2 (America, Europe and Oceania) | - | 1 (China) | 7 |
| c.393C>A | p.Asn131Lys | 3 (France) | 1 (USA) | - | - | - | - |
| c.392A>G | p.Asn131Ser | - | - | - | - | - | 1 |
| c.391A>T | p.Asn131Tyr | 1 (France) | - | - | - | - | - |
| c.394C>T | p.Pro132Thr | 1 (Czech) | - | - | - | - | - |
| c.395C>T | p.Pro132Leu | 1 (UK) | - | - | - | - | - |
| c.397C>G | p.Pro133Ala | - | - | 2 (America, Europe and Oceania) | - | - | - |
| c.400G>A | p.Asp134Asn | 6 (Belgium) | - | - | - | - | - |
| c.402C>A | p.Asp134Glu | 20  (Belgium 19,  Russia 1) | - | 2 (America, Europe and Oceania) | - | - | - |
| c.401A>G | p.Asp134Gly | 1 (Russia) | - | - | - | - | - |
| c.400G>C | p.Asp134His | - | - | 2 (America, Europe and Oceania) | - | - | - |
| c.405A>G | p.Ile135Met | - | - | - | - | 2 (Taiwan) | - |
| c.403A>C | p.Ile135Leu | 1 (Belgium) | - | - | - | - | 1 |
| c.404T>G | p.Ile135Arg | 1 (France) | - | - | - | - | - |
| c.404T>C | p.Ile135Thr | 2  (UK 1,  Russia 1) | 2 (USA) | 5 (America, Europe and Oceania) | - | - | - |
| c.407T>A | p.Val136Glu | - | 2 (USA) | - | - | - | - |
| c.410G>A | p.Gly137Asp | 3 (Norway) | - | 4 (America, Europe and Oceania) | - | - | - |
| c.409G>A | p.Gly137Ser | 1 (UK) | 4 (USA) | 5 (America, Europe and Oceania) | - | - | 2 |
| c.410G>T | p.Gly137Val | 1 (Italy) | - | - | - | - | - |
| c.414G>C | p.Lys138Asn | 1 (Russia) | - | - | - | - | - |
| c.416C>A | p.Thr139Asn | 1 (Russia) | - | - | - | - | - |
| c.419C>G | p.Ser140Cys | - | - | - | - | 2 (China) | - |
| c.418T>A | p.Ser140Thr | 1  (European  country) | 2 (USA) | 2 (America,  Europe and  Oceania) | - | - | - |
| c.418T>C | p.Ser140Pro | 1 (France) | - | - | - | - | - |
| c.421C>T | p.Gln141* | 4  (Switzerland 3,  European  country 1) | - | - | - | - | - |
| c.424G>T | p.Val142Phe | - | - | 2 (America,  Europe and  Oceania) | - | - | - |
| c.428C>G | p.Thr143Arg | 3 (Czech) | - | - | - | - | - |
| c.431delT | p.Leu144Argfs*18 | 1 (UK) | - | - | - | - | - |
| c.434A>C | p.Tyr145Ser | - | 6  (Costa Rica) | 3 (America,  Europe and  Oceania) | - | - | - |
| c.435T>A | p.Tyr145* | 4 (France) | - | - | - | - | - |
| c.433dupT | p.Tyr145Leufs*4 | 1 (Italy) | - | - | - | - | - |
| c.434_437del | p.Tyr145Serfs*16 | - | 1 (USA) | - | - | - | - |
| c.437T>C | p.Val146Ala | 1 (France) | - | - | - | - | - |
| c.437T>G | p.Val146Gly | 2 (Czech) | - | - | - | - | - |
| c.436G>T | p.Val146Phe | - | - | - | - | - | 1 |
| c.440T>C | p.Phe147Ser | - | - | - | - | 2 (China) | - |
| c.451C>A | p.Pro151Thr | - | 1 (USA) | - | - | - | - |
| c.462C>A | p.Tyr154* | 2 (Belgium) | 1 (USA) | - | - | - | - |
| c.449dupT | p.Pro151Alafs*3 | 3 (Italy) | - | - | - | - | - |
| c.486del | p.Ile162Metfs*90 | - | 1 (USA) | - | - | - | - |
| c.487G>A | p.Gly163Arg | 1 (Belgium) | 1 (USA) | - | - | - | 1 |
| c.487G>C | p.Gly163Arg | 4  (UK 2,  Netherlands 2) | 8 (USA) | - | - | - | - |
| c.496_499 delCTCG insTCC | p.Leu166Serfs*86 | 1 (UK) | - | - | - | - | - |
| c.499G>A | p.Gly167Arg | 2 (UK) | 1 (USA) | 2 (America,  Europe and  Oceania) | 1 (Kenya) | - | 1 |
| c.499C>A | p.Gly167Ala | 1 (UK) | - | - | - | - | - |
| c.509T>G | p.Leu170Arg | - | - | - | - | - | 2 |
| c.522_525 delGCTT | p.Leu175Serfs*76 | - | 2 (USA) | - | - | - | - |
| c.543C>G | p.Tyr181* | 3 (Belgium) | 1 (USA) | - | - | - | - |
| c.550_552 delCTAinsG | p.Leu184Alafs*50 | - | 1 (USA) | - | - | - | - |
| c.549dupG | p.Leu184Alafs*51 | 2  (Netherlands) | - | - | - | - | - |
| c.533delC | p.Arg185Alafs*67 | 1 (Austria) | - | - | - | - | - |
| c.554delG | p.Arg185Profs*67 | 1 (Spain) | - | - | - | - | - |
| c.560_566 delAGGCGGC | p.Gln187Profs*63 | - | - | - | - | 1 (Taiwan) | - |
| c.560delA | p.Gln187Argfs*65 | - | 1 (USA) | - | - | - | - |
| c.560_563 dupAGGC | p.Ala189Glyfs*47 | - | - | - | - | - | 1 |
| c.571C>T | p.Gln191* | - | 1 (USA) | - | - | - | - |
| c.570delG | p.Gln191Argfs*61 | 1 (Poland) | - | - | - | - | - |
| c.574_575del | p.Arg192Glufs*42 | - | 1 (USA) | - | - | - | - |
| c.588dupT | p.Met197Tyrfs*38 | 1 (Germany) | - | - | - | - | - |
| c.611A>T | p.Lys204Met | 7 (Spain) | - | - | - | - | - |
| c.616G>T | p.Gly206* | 3  (Germany 2,  European  country 1) | - | - | - | - | - |
| c.598_614 dup17 | p.Lys207Asnfs*51 | 1 (Spain) | - | - | - | - | - |
| c.626_630 delCGTCG | p.Ala209Glufs*24 | 1 (Belgium) | - | - | - | - | - |
| c.641G>A | p.Arg214Gln | - | - | 2 (America,  Europe and  Oceania) | - | - | - |
| c.643C>T | p.Gln215* | 2 (Italy) | 2 (USA) | 2 (America,  Europe and  Oceania) | - | - | 1 |
| c.646dupA | p.Thr216Asnfs*19 | - | 1 (USA) | 2 (America,  Europe and  Oceania) | - | - | - |
| c.649C>T | p.Pro217Ser | 1 (Germany) | - | - | - | - | - |
| c.661G>A | p.Ala221Thr | 1 (Turkey) | - | - | - | - | - |
| c.661_662 dupGC | p.Met222Glnfs*31 | 2 (Germany) | - | - | - | - | - |
| c.670G>T | p.Asp224Tyr | 9  (Germany 4,  Italy 4,  Austria 1) | - | - | - | - | - |
| c.674dupA | p.His225Glnfs*10 | - | - | - | - | 5 (China) | - |
| c.679A>G | p.Arg227Gly | - | - | - | - | - | 1 |
| c.681A>T | p.Arg227Ser | 2 (Serbia) | 1 (USA) | 2 (America,  Europe and  Oceania) | - | - | - |
| c.699_702 delTGAG | p.Ser233Argfs*18 | 5 (Italy) | - | - | - | 1 (Taiwan) | - |
| c.706A>G | p.Lys236Glu | - | - | - | - | 1 (Korea) | - |
| c.706_708 delAAG | p.Lys236del | - | 2 (USA) | 6 (America,  Europe and  Oceania) | - | - | - |
| c.745T>C | p.*249Gln | - | 1 (USA) | - | - | - | - |
| Duplication | - | - | 1 (USA) | - | - | - | - |
| 20.2kb incl. ex.1  & partial SDHC | - | - | 1 (USA) | - | - | - | - |
| Duplication incl. ex. 1-6 | - | - | 1  (Canada) | - | - | - | - |
| Duplication  118kb incl  entire gene  (5copies) | - | - | - | - | - | 6 (Taiwan) | - |
| Duplication  204kb incl  entire gene  & SDHC | - | - | 1  (Canada) | - | - | - | - |
| Duplication  4172bp incl  entire gene | - | 12  (Norway) | - | - | - | - | - |
| Duplication  4.2kb incl  entire coding  resion | - | - | 1 (USA) | - | - | - | - |
| T-C nt 341 I114T,  A-C nt 346 N116H,  G-A nt 382 D128N | - | - | 1 (USA) | - | - | - | - |
| c.-10_-6 dupTGCCC | - | 2 (Spain) | - | - | - | - | - |
| c.68-5_71del | - | - | 1 (USA) | - | - | - | - |
| c.235-2A>C | - | - | 1 (USA) | - | - | - | - |
| c.276G>A | - | 4 (France) | - | - | - | - | - |
| c.309G>T | - | 4 (Italy) | - | - | - | - | - |
| c.411C>T | - | 2 (Italy) | - | - | - | - | - |
| c.448+1G>A | - | - | 1 (USA) | - | - | - | - |
| c.448+2T>G | - | - | 1 (USA) | - | - | - | - |
| c.449-1G>A | - | - | 1 (USA) | - | - | - | - |
| c.449-1G>C | - | 2  (Spain 1,  Italy 1) | - | - | - | - | - |
| c.449-1G>T | - | - | - | - | - | 4 (Korea) | - |
| c.449-9C>T | - | 1 (Serbia) | - | - | - | - | - |
| c.584+2T>G | - | - | 3 (USA) | - | - | - | - |
| c.646-10_650del | - | - | 1 (USA) | - | - | - | - |
| c.645+1G>T | - | 1 (Czech) | - | - | - | - | - |
| c.675+3dupT | - | 1 (France) | - | - | - | - | - |

**Table S1**. Worldwide distribution and number of patients associated with *MPZ* variants. Table S1 is summarized to create Table 1.

| ID | Variant | Age | Onset | Inheritance pattern | Upper limb strength | Lower limb strength |
| --- | --- | --- | --- | --- | --- | --- |
| 3312 | p.Leu48Val | 57 | 49 | Sp | - | 2 |
| 3529 | p.Ser78Leu | 30 | 3 | Sp | - | - |
| 3542 | p.Thr124Met | 49 | 34 | AD | - | - |
| 3547 | p.Arg98Cys | 14 | 0 | Sp | 4 | - |
| 3640 | p.Arg98His | 28 | 15 | Sp | - | 4 |
| 3787 | p.Tyr82Cys | 5 | 3 | Sp | - | - |
| 3790 | p.Arg98Cys | 21 | 0 | Sp | - | 4 |
| 3999 | p.Asp128Asn | 55 | 0 | AD | 4 | 4 |
| 4075 | p.Asp75Val | 74 | 50 | AD | - | 4 |
| 4132 | p.Ile135Leu | 12 | - | - | - | - |
| 4201 | p.Tyr88His | 4 | 0 | Sp | - | - |
| 4274 | p.Val113Phe | 65 | - | - | - | - |
| 4276 | p.Asp61Asn | 7 | 0 | Sp | - | - |
| 4331 | p.Tyr82Cys | 3 | 1 | Sp | - | - |
| 4347 | p.Asn122Asp | 68 | 43 | AD | - | - |
| 4390 | p.Asp61Asn | 3 | 0 | Sp | 4 | 4 |
| 4407 | p.Trp101Arg | 22 | 6 | AD | 4 | 4 |
| 4442 | p.Arg98His | 50 | 36 | AD | - | 0 |
| 4554 | p.Arg98His | 29 | 24 | AD | - | 2 |
| 4613 | p.Arg98His | 36 | 6 | AD | - | 2 |
| 4673 | p.Thr124Met | 48 | 46 | AD | - | - |
| 4778 | p.Lys130Arg | 65 | 0 | AD | - | 4 |
| 4779 | p.Asp75Val | 71 | 52 | AD | - | 2 |
| 4793 | p.Arg98His | 43 | 40 | AD | - | 2 |
| 4815 | p.Ser78Leu | 5 | 1 | AD | - | - |
| 5037 | p.Asn122Asp | 47 | 42 | AD | - | - |
| 5058 | p.Ser78Leu | 53 | 40 | Sp | - | - |
| 5231 | p.Ile112Val | 78 | 58 | Sp | 4 | 4 |
| 5310 | p.Gly103Glu | 41 | 6 | AD | 1 | 4 |
| 5351 | p.Arg98His | 36 | 20 | Sp | - | 4 |
| 5476 | p.Arg98His | 49 | 43 | AD | 0 | 4 |
| 5506 | p.Gln215* | 27 | 5 | Sp | - | 4 |
| 5527 | p.Glu37Lys | 59 | 39 | AD | - | 4 |
| 5654 | p.Gly93Glu | 28 | 0 | AD | 4 | 2 |
| 5804 | p.Thr124Met | 55 | - | AD | - | - |
| 5861 | p.Gly137Ser | 12 | 3 | AD | - | - |
| 5886 | p.Thr124Met | 44 | 41 | Sp | - | - |
| 5895 | p.Arg98His | 73 | 66 | AD | - | 0 |
| 5898 | p.Thr124Met | 60 | 50 | AD | - | - |
| 5917 | p.Leu170Arg | 33 | 6 | AD | - | 4 |
| 5963 | p.Arg98His | 14 | 1 | Sp | - | - |
| 6024 | p.Thr124Met | 57 | 6 | AD | - | 4 |
| 6132 | p.His81Asp | 14 | 0 | AD | - | 2 |
| 6138 | p.Tyr68Cys | 77 | 71 | AD | - | - |
| 6181 | p.Asp75Gly | 59 | 57 | Sp | - | 1 |
| 6193 | p.Arg98Cys | 9 | 0 | Sp | - | - |
| 6258 | p.Asp75Val | 66 | 53 | AD | - | - |
| 6295 | p.Leu48Val | 69 | 64 | Sp | - | 1 |
| 6315 | p.Asp35Tyr | 61 | 54 | AD | - | - |
| 6428 | p.Arg98His | 29 | 1 | AD | 4 | 4 |
| 6533 | p.Arg98His | 47 | 31 | AD | - | - |
| 6542 | p.Arg98His | 33 | 0 | Sp | - | 4 |
| 6567 | p.Ile114Thr | 49 | 0 | AD | - | - |
| 6745 | p.Ile114Thr | 51 | 6 | AD | - | 2 |
| 6746 | p.Asp35Tyr | 60 | 55 | Sp | 0 | 4 |
| 6783 | p.Thr65Ile | 66 | 46 | AD | 3 | 1 |
| 6913 | p.Arg98His | 44 | 30 | - | 3 | 4 |
| 7031 | p.Lys130Arg | 57 | 27 | Sp | 3 | 4 |
| 7078 | p.Thr34Ile | 27 | 1 | Sp | - | 4 |
| 7163 | p.Val142Asp | 53 | 43 | AD | 4 | 4 |
| 7181 | p.Arg98His | 41 | 35 | Sp | - | 2 |
| 7199 | p.His81Asp | 12 | 0 | AD | - | 1 |
| 7240 | p.Arg98His | 31 | 3 | AD | - | 4 |
| 7344 | p.Asp75Val p.Phe19Ser | 77 | 30 | AD | 0 | 3 |
| 7389 | p.Ser111Tyr | 23 | 0 | Sp | - | - |
| 7433 | p.Arg98His | 55 | 53 | Sp | - | - |
| 7468 | p.Asp75Val | 47 | 45 | AD | - | - |
| 7484 | p.Phe19Ser | 56 | 51 | Sp | - | 0 |
| 7542 | p.Asp35Tyr | 75 | 55 | Sp | 1 | 0 |
| 7552 | p.Arg98His | 75 | 74 | Sp | 0 | 0 |
| 7743 | p.Asp75Val | 57 | 40 | AD | - | - |
| 8031 | p.Arg98Cys | 3 | 0 | Sp | - | - |
| 8045 | p.Arg98His | 40 | 10 | AD | 4 | 4 |
| 8149 | p.Arg98His | 49 | 20 | Sp | - | 4 |
| 8160 | p.Ser54Tyr | 2 | 0 | Sp | - | - |
| 8202 | p.Arg98His | 69 | 60 | Sp | - | 0 |
| 8210 | p.Gly137Ser | 45 | 44 | Sp | - | - |

(continued below)

| ID | Variant | Median MCV | Demyelinating/Axonal CMT | CK (U/L) | CSF protein (mg/dL) |
| --- | --- | --- | --- | --- | --- |
| 3312 | p.Leu48Val | - | U | 589 | 32.5 |
| 3529 | p.Ser78Leu | 18.5 | D | 74 | 40 |
| 3542 | p.Thr124Met | - | U | - | - |
| 3547 | p.Arg98Cys | 31.2 | D | 78 | - |
| 3640 | p.Arg98His | 15.1 | D | 123 | 125 |
| 3787 | p.Tyr82Cys | - | U | - | - |
| 3790 | p.Arg98Cys | - | U | - | 94 |
| 3999 | p.Asp128Asn | 14.6 | D | 204 | 67 |
| 4075 | p.Asp75Val | 44.8 | A | - | - |
| 4132 | p.Ile135Leu | - | U | - | - |
| 4201 | p.Tyr88His | 10 | D | - | 56 |
| 4274 | p.Val113Phe | - | U | - | - |
| 4276 | p.Asp61Asn | 5.1 | D | - | - |
| 4331 | p.Tyr82Cys | 19 | D | 113 | 30 |
| 4347 | p.Asn122Asp | 31 | D | 297 | 108 |
| 4390 | p.Asp61Asn | 4 | D | 166 | 54 |
| 4407 | p.Trp101Arg | - | U | 156 | 100 |
| 4442 | p.Arg98His | 20.4 | D | 76 | 106 |
| 4554 | p.Arg98His | 17 | D | - | - |
| 4613 | p.Arg98His | 19 | D | 235 | 63 |
| 4673 | p.Thr124Met | - | U | - | - |
| 4778 | p.Lys130Arg | 12.6 | D | - | - |
| 4779 | p.Asp75Val | <38 | D | - | - |
| 4793 | p.Arg98His | 25.7 | D | - | - |
| 4815 | p.Ser78Leu | 14 | D | 96 | - |
| 5037 | p.Asn122Asp | 39 | A | - | - |
| 5058 | p.Ser78Leu | 20 | D | 174 | 28 |
| 5231 | p.Ile112Val | 51.3 | A | - | 115 |
| 5310 | p.Gly103Glu | 8 | D | - | - |
| 5351 | p.Arg98His | 15.4 | D | - | - |
| 5476 | p.Arg98His | - | U | 353 | 96 |
| 5506 | p.Gln215* | 3.9 | D | - | - |
| 5527 | p.Glu37Lys | 55.9 | A | - | 33 |
| 5654 | p.Gly93Glu | 17.3 | D | - | - |
| 5804 | p.Thr124Met | - | U | - | - |
| 5861 | p.Gly137Ser | 10.6 | D | 312 | - |
| 5886 | p.Thr124Met | - | U | - | - |
| 5895 | p.Arg98His | 23.6 | D | 400 | 73 |
| 5898 | p.Thr124Met | 39.3 | A | - | - |
| 5917 | p.Leu170Arg | - | U | - | - |
| 5963 | p.Arg98His | 15.4 | D | - | - |
| 6024 | p.Thr124Met | 50.7 | A | - | 32 |
| 6132 | p.His81Asp | 11.3 | D | 170 | 64 |
| 6138 | p.Tyr68Cys | 30.9 | D | - | - |
| 6181 | p.Asp75Gly | 47 | A | 326 | 46 |
| 6193 | p.Arg98Cys | - | U | - | - |
| 6258 | p.Asp75Val | 47.7 | A | - | - |
| 6295 | p.Leu48Val | 44.7 | A | 131 | 26.2 |
| 6315 | p.Asp35Tyr | 49.8 | A | - | - |
| 6428 | p.Arg98His | 13.3 | D | - | - |
| 6533 | p.Arg98His | 18 | D | - | - |
| 6542 | p.Arg98His | 12 | D | - | - |
| 6567 | p.Ile114Thr | 38 | A | - | - |
| 6745 | p.Ile114Thr | 37.9 | D | - | - |
| 6746 | p.Asp35Tyr | 44.1 | A | 621 | - |
| 6783 | p.Thr65Ile | - | U | 183 | 51 |
| 6913 | p.Arg98His | 19 | D | - | - |
| 7031 | p.Lys130Arg | - | U | - | - |
| 7078 | p.Thr34Ile | 6 | D | - | - |
| 7163 | p.Val142Asp | 16 | D | - | - |
| 7181 | p.Arg98His | 15.1 | D | 1000< | - |
| 7199 | p.His81Asp | 9.1 | D | - | - |
| 7240 | p.Arg98His | 10.3 | D | - | 136 |
| 7344 | p.Asp75Val p.Phe19Ser | 31.1 | D | - | 45 |
| 7389 | p.Ser111Tyr | 3.8 | D | - | - |
| 7433 | p.Arg98His | 21.1 | D | 174 | - |
| 7468 | p.Asp75Val | 54.1 | A | - | - |
| 7484 | p.Phe19Ser | 40.5 | A | 89 | 52 |
| 7542 | p.Asp35Tyr | 51.1 | A | 153 | 25.3 |
| 7552 | p.Arg98His | 23 | D | 96 | 49 |
| 7743 | p.Asp75Val | 44 | A | - | - |
| 8031 | p.Arg98Cys | - | U | 218 | 45 |
| 8045 | p.Arg98His | 9.8 | D | 82 | - |
| 8149 | p.Arg98His | 24 | D | - | - |
| 8160 | p.Ser54Tyr | - | U | 189 | 72 |
| 8202 | p.Arg98His | 21.7 | D | - | - |
| 8210 | p.Gly137Ser | 13.8 | D | 132 | 64 |

**Table S2.** Clinical data of 77 CMT patients with *MPZ* variants in this study. Abbreviations: AD, autosomal dominant ; Sp, Sporadic ; Upper limb strength, Upper limb strength scores in CMT neuropathy scores ; Lower limb strength, Lower limb strength scores in CMT neuropathy scores ; CK, creatine kinase ; CSF, cerebrospinal fluid ; D, demyelinating CMT ; A, axonal CMT ; U, unclassified type ; - , Not available or not evoked

| ID | *MPZ* variants | Cranial nerve involvement |
| --- | --- | --- |
| 3542 | p.Thr124Met | Anisocoria |
|  |  | Adie's pupil |
| 3547 | p.Arg98Cys | Involuntary movement of tongue |
| 3999 | p.Asp128Asn | Dysphagia |
|  |  | Dysarthria |
|  |  | Atrophy of tongue |
|  |  | Tongue fasciculation |
| 4390 | p.Asp61Asn | Weakness of facial muscle |
|  |  | Atrophy of Trapezius and Sternocleidomastoid |
| 4554 | p.Arg98His | Atrophy of facial muscle |
| 4613 | p.Arg98His | Weakness of facial muscle |
|  |  | Tinnitus of right ear |
|  |  | Deviation of tongue protrusion |
| 4793 | p.Arg98His | Deviation of tongue protrusion |
| 5231 | p.Ile112Val | Bilateral ptosis |
| 5310 | p.Gly103Glu | Hearing loss |
| 5917 | p.Leu170Arg | Right strabismus |
|  |  | Right facial nerve paralysis |
|  |  | Right hearing loss |
|  |  | Deviation of tongue protrusion |
|  |  | Atrophy of tongue |
| 6258 | p.Asp75Val | Slight dysphagia |
|  |  | Dysarthria |
| 6295 | p.Leu48Val | Anisocoria |
|  |  | Sluggish light reflex |
|  |  | Hearing loss |
|  |  | Dysarthria |
| 6745 | p.Ile114Thr | Pain in the trigeminal nerve region |
| 6746 | p.Asp35Tyr | Atrophy of facial muscle |
| 6913 | p.Arg98His | Nystagmus |
| 7031 | p.Lys130Arg | Right strabismus |
|  |  | Hearing loss |
| 7344 | p.Asp75Val p.Phe19Ser | Anisocoria |
|  |  | Nystagmus |
|  |  | Slight tremulous speech |
| 7552 | p.Arg98His | Dysarthria |
|  |  | Dysphagia |
| 8031 | p.Arg98Cys | Dysarthria |
| 8045 | p.Arg98His | Weakness of facial muscle |
|  |  | Dysarthria |
|  |  | Dysphagia |

**Table S3**. Patients with cranial nerve involvement.

| Electrophysiological classification | CK elevated | CK normal | p-value |
| --- | --- | --- | --- |
|  | (n = 6) | (n = 18) |  |
| Axonal  type | 2 | 3 | 0.568 |

**Table S4**. Numbers of patients with axonal type in elevated and normal CK groups.

| ID | Mutation | CSF protein(mg/dL) | MRI findings |
| --- | --- | --- | --- |
| 7484 | p.Phe19Ser | 52 | Slight enlargement of cauda equina |
| 8160 | p.Ser54Tyr | 72 | - |
| 4390 | p.Asp61Asn | 54 | Not particular findings |
| 6783 | p.Thr65Ile | 51 | Spinal canal stenosis |
| 6132 | p.His81Asp | 64 | Not particular findings |
| 4201 | p.Tyr88His | 56 | - |
| 3790 | p.Arg98Cys | 94 | Enlargement of ventral spinal nerve root |
| 4613 | p.Arg98His | 63 | - |
| 5895 | p.Arg98His | 73 | Spinal canal stenosis |
| 5476 | p.Arg98His | 96 | Spinal canal stenosis |
| 4442 | p.Arg98His | 106 | - |
| 3640 | p.Arg98His | 125 | - |
| 7240 | p.Arg98His | 136 | - |
| 4407 | p.Trp101Arg | 100 | - |
| 5231 | p.Ile112Val | 115 | - |
| 4347 | p.Asn122Asp | 108 | - |
| 3999 | p.Asp128Asn | 67 | - |
| 8210 | p.Gly137Ser | 64 | Findings of cervical spondylosis |

**Table S5**. MRI findings of patients with elevated CSF protein. - : Not available

Abbreviations: - ; Not available

【Referenced reports related to *MPZ* variants in Human Genome Mutation Database】

1. DiVincenzo C, Rlzinga CD, Medeiros AC, *et al.* The allelic spectrum of Charcot-Marie-Tooth disease in over 17,000 individuals with neuropathy. *Mol Genet Genomic Med* 2014; **2**: 522-529.

2. Finsterer J, Miltenberger G, Rauschka H, Janecke A. Novel C59T leader peptide mutation in the MPZ gene associated with late-onset, axonal, sensorimotor polyneuropathy. *Eur J Neurol* 2006; **13**: 1149-1152.

3. Milley GM, Varga ET, Grosz Z, *et al.* Genotypic and phenotypic spectrum of the most common causative genes of Charcot-Marie-Tooth disease in Hungarian patients. *Neuromuscul Disord* 2018; **28**: 38-42.

4. Niermeijer JMF, Jansweijer M, van der Kooi A, *et al.* A novel pathogenic mutation of the MPZ gene causing hereditary neuropathy. *Neuromuscul Disord* 2011; **11**: 688.

5. Miltenberger-Miltenyi G, Schwarzbraun T, Loscher WN, *et al.* Identification and in silico analysis of 14 novel GJB1, MPZ and PMP22 gene mutations. *Eur J Hum Genet* 2009; **17**: 1154-1159.

6. Floroskufi P, Panas M, Karadima G, Vassilopoulos D. New mutation of the MPZ gene in a family with the Dejerine-Sottas disease phenotype. *Muscle Nerve* 2007; **35**: 667-669.

7. Yoshihara T, Yamamoto M, Doyu Manabu, *et al.* Mutations in the peripheral myelin protein zero and connexin32 genes detected by non-isotopic RNase cleavage assay and their phenotypes in Japanese patients with Charcot-Marie-Tooth disease. *Hum Mutat* 2000; **16**: 177-178.

8. Chen CX, Dong HL, Wei Q, et al. Genetic spectrum and clinical profiles in a southeast Chinese cohort of Charcot-Marie-Tooth disease. *Clin Genet* 2019; **96**: 439-448.

9. Gabreels-Fasten AA, Hoogendijk JE, Meijerink PH, *et al.* Two divergent types of nerve pathology in patients with different P0 mutations in Charcot-Marie-Tooth disease. *Neurology* 1996; **47**: 761-765.

10. Braathen GJ, Sand JC, Russell MB. Two novel missense mutations in the myelin protein zero gene causes Charcot-Marie-Tooth type 2 and Dejerine-Sottas syndrome. *BMC Res Notes* 2010; **3**: 99.

11. Ostern R, Fagerheim T, Hjellnes H, Nygard B, Mellgren SI, Nilssen O. Segregation analysis in families with Charcot-Marie-Tooth disease allows reclassification of putative disease causing mutations. *BMC Med Genet* 2014; **15**: 12.

12. Mastaglia FL, Nowak KJ, Stell R, *et al.* Novel mutation in the myelin protein zero gene in a family with intermediate hereditary motor and sensory neuropathy. *J Neurol Neurosurg Psychiatry* 1999; **67**: 174-179.

13. Dacci P, Taroni F, Bella ED *et al.* Myelin protein zero Arg36Gly mutation with very late onset and rapidly progressive painful neuropathy. *J Peripher Nerv Syst* 2012; **17**: 422-425.

14. Bruns TM, Phillips LH, Dimberg EL, Vaught BK, Klein CJ. Novel myelin protein zero mutation (Arg36Trp) in a patient with acute onset painful neuropathy. *Neuromuscul Disord* 2006; **16**: 308-310.

15. Shy ME, Jani A, Krajewski K, *et al.* Phenotypic clustering in MPZ mutations. *Brain* 2004; **127**: 371-384.

16. Kilfoyle DH, Dyck PJ, Wu Y, et al. Myelin protein zero mutation His39Pro: hereditary motor and sensory neuropathy with variable onset, hearing loss, restless legs and multiple sclerosis. *J Neurol Neurosurg Psychiatry* 2006; **77**: 963-966.

17. Marrosu MG, Vaccargiu S, Marrosu G, Vannelli A, Cianchetti C, Muntoni F. Charcot-Marie-Tooth disease type 2 associated with mutation of the myelin protein zero gene. *Neurology* 1998; **50**: 1397-1401.

18. Lorefice L, Murru MR, Coghe G, et al. Charcot-Marie-Tooth disease: genetic subtypes in the Sardinian population. *Neurol Sci* 2017; **38**: 1019-1025.

19. Brozkova D, Mazanec R, Haberlova J, Sakmaryova I, Seeman P. Clinical and in silico evidence for and against pathogenicity of 11 new mutations in the MPZ gene. *Clin Genet* 2010; **78**: 81-87.

20. Szabo A, Zuchner S, Siska E, Mechler F, Molnar MJ. Marked phenotypic variation in a family with a new myelin protein zero mutation. *Neuromuscul Disord* 2005; **15**: 760-763.

21. Nishiyama S, Sugeno N, Tateyama M, Aoki M. Late-onset Charcot-Marie-Tooth disease type 1B due to a novel mutation in the extracellular disulfide bridge of MPZ gene. *Clin Neurol Neurosurg* 2013; **115**: 208-209.

22. Antoniadi T, Buxton C, Dennis G, *et al.* Application of targeted multi-gene panel testing for the diagnosis of inherited peripheral neuropathy provides a high diagnostic yield with unexpected phenotype-genotype variability. *BMC Med Genet* 2015; **16**: 84.

23. Young P, Grote K, Kuhlenbaumer G, *et al.* Mutation analysis in Chariot-Marie Tooth disease type 1: point mutations in the MPZ gene and the GJB1 gene cause comparable phenotypic heterogeneity. *J Neurol* 2001; **248**: 410-415.

24. Nam SH, Hong YB, Hyun YS, *et al.* Identification of Genetic Causes of Inherited Peripheral Neuropathies by Targeted Gene Panel Sequencing. *Mol Cells* 2016; **39**: 382-338.

25. Vaeth S, Christensen R, Duno M, *et al.* Genetic analysis of Charcot-Marie-Tooth disease in Denmark and the implementation of a next generation sequencing platform. *Eur J Med Genet* 2019; **62**: 1-8.

26. Hoyer H, Braathen GJ, Busk OL, *et al.* Genetic diagnosis of Charcot-Marie-Tooth disease in a population by next-generation sequencing. *Biomed Res Int* 2014; **2014**: 210401.

27. Baissar-Tadmouri N, Gulsen-Parman Y, Latour P, *et al.* Two novel mutations in the MPZ gene coding region in Charcot-Marie-Tooth type 1 patients of Turkish origin: S54P, [I30del; GVYI29ins]. *Hum Mutat* 1999; **14**: 499.

28. Hoebeke C, Bonello-Palot N, Audic F, *et al.* Retrospective study of 75 children with peripheral inherited neuropathy: Genotype-phenotype correlations. *Arch Pediatr* 2018; **25**: 452-458.

29. Kleffner I, Schirmacher A, Gess B, Boentert M, Young P. Four novel mutations of the myelin protein zero gene presenting as a mild and late-onset polyneuropathy. *J Neurol* 2010; **257**: 1864-1868.

30. Kochanski A, Kabzinska D, Nowakowski A, Drac H, Hausmanowa-Petrusewicz I. An axonal form of Charcot-Marie-Tooth disease with a novel missense mutation in the myelin protein zero gene. *J Peripher Nerv Syst* 2004; **9**: 1-2.

31. Lee YC , Soong BW, Lin KP, Lee HY, Wu ZA, Kao KP. Myelin protein zero gene mutations in Taiwanese patients with Charcot-Marie-Tooth disease type 1. *J Neurol Sci* 2004; **219**: 95-100.

32. Sorour E, Upadhyaya M. Mutation analysis in Charcot-Marie-Tooth disease type 1 (CMT1). *Hum Mutat* 1998; **Suppl 1**: S242-247.

33. Auer-Grumbach M , Strasser-Fuchs S, Robl T, Windpassinger C, Wagner K. Late onset Charcot-Marie-Tooth 2 syndrome caused by two novel mutations in the MPZ gene. *Neurology* 2003; **61**: 1435-1437.

34. Bellone E, Cassandrini D, Di Maria E, *et al.* Novel MPZ mutation in a sporadic CMT patient. *J Peripher Nerv Syst* 2001; **6**: 40-59.

35. Yonekawa T, Komaki H, Saito Y, Takashima H, Masayuki S. Congenital hypomyelinating neuropathy attributable to a de novo p.Asp61Asn mutation of the myelin protein zero gene. *Pediatr Neurol* 2013; **48**: 59-62.

36. Senderek J, Hermanns B, Lehmann U, *et al.* Charcot-Marie-Tooth neuropathy type 2 and P0 point mutations: two novel amino acid substitutions (Asp61Gly; Tyr119Cys) and a possible "hotspot" on Thr124Met. *Brain Pahol* 2000; **10**: 235-248.

37. Nakagawa M, Suehara M, Saito A, *et al.* A novel MPZ gene mutation in dominantly inherited neuropathy with focally folded myelin sheaths. *Neurology* 1999; **52**: 1271-1275.

38. Blanquet-Grossard F, Pham-Dinh D, Dautigny A, Latour P, *et al.* Charcot-Marie-Tooth type 1B neuropathy: third mutation of serine 63 codon in the major peripheral myelin glycoprotein PO gene. *Clin Genet* 1995; **48**: 281-283.

39. Kochanski A, Drac H, Kabzinska D, Hausmanowa-Petrusewicz I. A novel mutation, Thr65Ala, in the MPZ gene in a patient with Charcot-Marie-Tooth type 1B disease with focally folded myelin. *Neuromuscul Disord* 2004; **14**: 229-232.

40. Nadol JB, Hedley-Whyte ET, Amr SS, O’Malley JT, Kamakura T. Histopathology of the Inner Ear in Charcot-Marie-Tooth Syndrome Caused by a Missense Variant (p.Thr65Ala) in the MPZ Gene. *Audiol Neurol* 2018; **23**: 326-334.

41. Numakura C, Lin C, Ikegami T, Guldberg P, Hayasaka K. Molecular analysis in Japanese patients with Charcot-Marie-Tooth disease: DGGE analysis for PMP22, MPZ, and Cx32/GJB1 mutations. *Hum Mutat* 2002; **20**: 392-398.

42. Young T, Shuey N, Partridge J, Bremner FD, Nicholl DJ. Compound Charcot-Marie-Tooth disease: a kindred with severe hereditary neuropathy, pupil abnormalities and a novel MPZ mutation. *J Neurol Neurosurg Psychiatry* 2013; **84**: 234-236.

43. Hisama FM. Familial periodic paralysis and Charcot-Marie-Tooth disease in a 7-generation family. *Arch Neurol* 2005; **62**: 135-138.

44. Khadilkar SV, Patil ND, Kadam ND, Mansukhani KA, Patel BA. Clinico-Electrophysiological and Genetic Overlaps and Magnetic Resonance Imaging Findings in Charcot-Marie- Tooth Disease: A Pilot Study from Western India. *Ann Indian Acad Neurol* 2017; **20**: 425-429.

45. Anandh U, Nikalji R, Parick A. Membranous Nephropathy in a Patient with Charcot-Marie-Tooth Disease: Association of Myelin Mutations. *India J Nephrol* 2018; **28**: 397-400.

46. Nicolaou P, Zamba-Papanicolaou E, Koutsou P, et al. Charcot-Marie-Tooth disease in Cyprus: epidemiological, clinical and genetic characteristics. Neuroepidemiology 2010; 35: 171-177.

47. Laura M, Milani M, Morbin M, *et al.* Rapid progression of late onset axonal Charcot-Marie-Tooth disease associated with a novel MPZ mutation in the extracellular domain. *J Neurol Neurosurg Psychiatry* 2007; **78**: 1263-1266.

48. Lagueny A, Latour P, Vital A, *et al.* Mild recurrent neuropathy in CMT1B with as novel nonsense mutation in the extracellular domain of the MPZ gene. *J Neurol Neurosurg Psychiatry* 2001; **70**: 232-235.

49. Misu K, Yoshihara T, Shikama Y, *et al*. An axonal form of Charcot-Marie-Tooth disease showing distinctive features in association with mutations in the peripheral myelin protein zero gene (Thr124Met or Asp75Val). *J Neurol Neurosurg Psychiatry* 2000; **69**: 806-811.

50. Nelis E, Timmerman V, De Jonghe P, *et al.* Rapid screening of myelin genes in CMT1 patients by SSCP analysis: identification of new mutations and polymorphisms in the P0 gene. *Hum Genet* 1994; **94**: 653-657.

51. Fabrizi GM, Taioli F, Cavallaro T, *et al.* Focally folded myelin in Charcot-Marie-Tooth neuropathy type 1B with Ser49Leu in the myelin protein zero. *Acta Neuropathol* 2000; **100**: 299-304.

52. Benko WS, Hruska KS, Nagan N, *et al.* Uniparental disomy of chromosome 1 causing concurrent Charcot-Marie-Tooth and Gaucher disease Type 3. *Neurology* 2008; **70**: 976-978.

53. Kakar R, Ma W, Dutra A, Seltzer WK, Grewal RP. Clinical and genetic analysis of CMT1B in a Nigerian family. *Muscle Nerve* 2003; **27**:628-630.

54. Sorour E, MacMillan J, Upadhyaya M. Novel mutation of the myelin P0 gene in a CMT1B family. *Hum Mutat* 1997; **9**:74-77.

55. Beats J, Deconinck T, De Vriendt E, *et al.* Genetic spectrum of hereditary neuropathies with onset in the first year of life. *Brain* 2011; **134**: 2664-2676.

56. Choi BO, Kim SB, Kanwal S, *et al.* MPZ mutation in an early-onset Charcot-Marie-Tooth disease type 1B family by genome-wide linkage analysis. *Int J Mol Med* 2011; **28**:389-396.

57. Liu L, Li X, Zi X, *et al*. Two novel MPZ mutations in Chinese CMT patients. *J Peripher Nerv Syst* 2013 ; **18**: 256-260.

58. Bienfait HM, Baas F, Gabreëls-Festen AA, Koelman JH, Langerhorst CT, de Visser M. Two amino-acid substitutions in the myelin protein zero gene of a case of Charcot-Marie-Tooth disease associated with light-near dissociation. *Neuromuscul Disord* 2002; **12**: 281-285.

59. Silander K, Meretoja P, Juvonen V, *et al.* Spectrum of mutations in Finnish patients with Charcot-Marie-Tooth disease and related neuropathies. *Hum Mutat* 1998; **12**: 59-68.

60. Bienfait HME , Faber CG, Baas F, *et al.* Late onset axonal Charcot-Marie-Tooth phenotype caused by a novel myelin protein zero mutation. *J Neurol Neurosurg Psychiatry* 2006; **77**: 534-537.

61. Manganelli F, Tozza S, Pisciotta C, *et al.* Charcot-Marie-Tooth disease: frequency of genetic subtypes in a Southern Italy population. *J Peripher Nerv Syst* 2014; **19**: 292-298.

62. Dohrn MF, Glockle N, Mulahasanovic L, *et al.* Frequent genes in rare diseases: panel-based next generation sequencing to disclose causal mutations in hereditary neuropathies. *J Neurochem* 2017; **143**: 353-359.

63. Boerkoel CF, Takashima H, Garcia CA, *et al.* Charcot-Marie-Tooth disease and related neuropathies: mutation distribution and genotype-phenotype correlation. *Ann Neurol* 2002 ; **51**: 190-201.

64. Lupo V, Pascual-Pascual SI, Sancho P, *et al.* Complexity of the Hereditary Motor and Sensory Neuropathies: Clinical and Cellular Characterization of the MPZ p.D90E Mutation. *J Child Neurol* 2015; **30**:1544-1548.

65. Ikegami T, Ikeda H, Mitsui T, Hayasaka K, Ishii S. Novel mutation of the myelin Po gene in a pedigree with Charcot-Marie-Tooth disease type 1B. *Am J Med Genet* 1997; **71**: 246-248.

66. Sun Y, Man J, Wan Y, *et al.* Targeted next-generation sequencing as a comprehensive test for Mendelian diseases: a cohort diagnostic study. *Sci Rep* 2018; **8**: 11646.

67. Seeman P, Mazanec R, Huehne K, Suslikova P, Kellar O, and Rautenstrauss B. Hearing loss as the first feature of late-onset axonal CMT disease due to a novel P0 mutation. *Neurology* 2004; **63**: 733-735.

68. Rouger H, LeGuern E, Gouider R, *et al.* High frequency of mutations in codon 98 of the peripheral myelin protein P0 gene in 20 French CMT1 patients. *Am J Hum Genet* 1996; **58** : 638-41.

69. Phillips JP, Warner LE, Lupski JR, Garg BP. Congenital hypomyelinating neuropathy: two patients with long-term follow-up. *Pediatr Neurol* 1999; **20**: 226-232.

70. Ohnishi A, Yamamoto T, Yamamori S, Sudo K, Fukushima Y, Ikeda M. Myelinated fibers in Charcot-Marie-Tooth disease type 1B with Arg98His mutation of Po protein. *J Neurol Sci* 1999; **171**: 97-109.

71. Wang W, Wang C, Dawson DB, *et al.* Target-enrichment sequencing and copy number evaluation in inherited polyneuropathy. *Neurology* 2016; **86**: 1762-1771.

72. Warner LE, Hilz MJ, Appel SH, *et al.* Clinical phenotypes of different MPZ (P0) mutations may include Charcot-Marie-Tooth type 1B, Dejerine-Sottas, and congenital hypomyelination. *Neuron* 1996; **17**: 451-460.

73. Donaghy M, Sisodiya SM, Kennett R, McDonald B, Haites N, Bell C*.* Steroid responsive polyneuropathy in a family with a novel myelin protein zero mutation. *J Neurol Neurosurg Psychiatry* 2000; **69**: 799–805.

74. Fabrizi GM, Ferrarini M, Cavallaro T, Jarre L, Polo A, Rizzuto N. A somatic and germline mosaic mutation in MPZ/P(0) mimics recessive inheritance of CMT1B. *Neurology* 2001; **57**: 101-105.

75. Kabzinska D, Korwin-Piotrowska T, Drechsler H, Drac H, Hausmanowa-Petrusewicz I, Kochanski A. Late-onset Charcot-Marie-Tooth type 2 disease with hearing impairment associated with a novel Pro105Thr mutation in the MPZ gene. *Am J Med Genet A* 2007; **143A**: 2196-2199.

76. Marttila M, Rautenstrauss B, Huehne K, Laitinen V, Majamaa K, Karppa M. A novel mutation of myelin protein zero associated with late-onset predominantly axonal Charcot-Marie-Tooth disease. *J Neurol* 2012; **259**:1585-1589.

77. Lagueny A, Latour P, Vital A, *et al.* Peripheral myelin modification in CMT1B correlates with MPZ gene mutations. *Neuromuscul Disord* 1999; **9**: 361-367.

78. Santoro L, Manganelli F, Maria ED, *et al.* A novel mutation of myelin protein zero associated with an axonal form of Charcot-Marie-Tooth disease. *J Neurol Neurosurg Psychiatry* 2004; **75**: 262-265.

79. Mandich P, Fossa P, Capponi S, *et al*. Clinical features and molecular modelling of novel *MPZ* mutations in demyelinating and axonal neuropathies. *Eur J Hum Genet* 2009; **17**: 1129–1134.

80. Sevilla T, Lupo V, Sivera R, *et al.* Congenital hypomyelinating neuropathy due to a novel MPZ mutation. *J Peripher Nerv Syst* 2011; **16**: 347-352.

81. Murphy SM, Laura M, Blake J, Polke J, Bremner F, Reilly MM. Conduction block and tonic pupils in Charcot-Marie-Tooth disease caused by a myelin protein zero p.Ile112Thr mutation. *Neuromuscul Disord* 2011; **21**: 223-226.

82. Takashima H, Boerkoel CF, Lupski JR, *et al.* Screening for mutations in a genetically heterogeneous disorder: DHPLC versus DNA sequence for mutation detection in multiple genes causing Charcot-Marie-Tooth neuropathy. *Genet Med* 2001; **3**: 335-342.

83. Choi BO, Lee MS, Shin SH, *et al.* Mutational analysis of PMP22, MPZ, GJB1, EGR2 and NEFL in Korean Charcot-Marie-Tooth neuropathy patients. *Hum Mutat* 2004; **24**: 185-186.

84. Duan X, Gu W, Hao Y, *et al.* A Novel Asp121Asn Mutation of Myelin Protein Zero Is Associated with Late-Onset Axonal Charcot-Marie-Tooth Disease, Hearing Loss and Pupil Abnormalities. *Front Aging Neurosci* 2016; **8**: 222.

85. Blanquet-Grossard F, Pham-Dinh D, Dautigny A, *et al*. Charcot-Marie-Tooth type 1B neuropathy: a mutation at the single glycosylation site in the major peripheral myelin glycoprotein Po. *Hum Mutat* 1996; **8**: 185-186.

86. Lee YC, Yu CTR, Lin KP, *et al.* MPZ mutation G123S characterization: evidence for a complex pathogenesis in CMT disease. *Neurology* 2008; **70**: 273-277.

87. Kochanski A, Drac H, Kabzinska D, *et al.* A novel MPZ gene mutation in congenital neuropathy with hypomyelination. *Neurology* 2004; **62**: 2122-2123.

88. Schiavon F, Rampazzo A, Merlini L, Angelini C, Mostacciuolo ML. Mutations of the same sequence of the myelin P0 gene causing two different phenotypes. *Hum Mutat* 1998; **Suppl 1**: S217-219.

89. Misu K, Yoshihara T, Yamamoto M, *et al.* Two families of Charcot-Marie-Tooth disease with Adie’s pupil, axonal neuropathy and the Thr124Met mutation in the peripheral myelin protein zero gene. *Rinsho Shinkeigaku* 2000; **40**: 149-154.

90. Baloh RH, Jen JC, Kim G, Baloh RW. Chronic cough due to Thr124Met mutation in the peripheral myelin protein zero (MPZ gene). *Neurology* 2004; **62**: 1905-1906.

91. Sanmaneechai O, Feely S, Scherer SS, *et al.* Genotype–phenotype characteristics and baseline natural history of heritable neuropathies caused by mutations in the *MPZ* gene. *Brain* 2015; **138**: 3180-3192.

92. Farwell KD, Shahmirzadi L, El-Khechen D, *et al.* Enhanced utility of family-centered diagnostic exome sequencing with inheritance model-based analysis: results from 500 unselected families with undiagnosed genetic conditions. *Genet Med* 2015; **17**: 578-586.

93. Fabrizi GM, Cavallaro T, Morbin M, Simonati A, Taioli F, Rizzuto N. Novel mutation of the P0 extracellular domain causes a Déjérine-Sottas syndrome. *J Neurol Neurosurg Psychiatry* 1999; **66**: 386-389.

94. Marques W, Hanna MG, Marques SR, Sweeney MG, Thomas PK, Wood NW. Phenotypic variation of a new P0 mutation in genetically identical twins. *J Neurol* 1999; **246**: 596-599.

95. Houlden H, Reilly MM, Smith S. Pupil abnormalities in 131 cases of genetically defined inherited peripheral neuropathy. *Eye (Lond)* 2009; **23**: 966-974.

96. Tachi N, Kozuka N, Ohya K, *et al.* A new mutation of the Po gene in patients with Charcot-Marie-Tooth disease type 1B: screening of the Po gene by heteroduplex analysis. *Neurosci Lett* 1996; **204**: 173-176.

97. Plante-Bordeneuve V, Guiochon-Mantel A, Lacroix C, Lapresle J, Said G. The Roussy-Lévy family: from the original description to the gene. *Ann Neurol* 1999; **46**: 770-773.

98. McMillan HJ, Santagata S, Shapiro F, *et al.* Novel MPZ mutations and congenital hypomyelinating neuropathy. *Neromuscul Disord* 2010; **20**: 725-729.

99. Iida M, Koike H, Ando T, *et al.* A novel MPZ mutation in Charcot-Marie-Tooth disease type 1B with focally folded myelin and multiple entrapment neuropathies. *Neuromuscul Disord* 2012; **22**: 166-169.

100. Vallat JM, Magy L, Lagrange E, *et al.* Diagnostic value of ultrastructural nerve examination in Charcot-Marie-Tooth disease: two CMT 1B cases with pseudo-recessive inheritance. *Acta Neuropathol* 2007; **113**: 443-449.

101. Nelis E, Timmerman V, De Jonghe P, Muylle L, Martin JJ, Van Broeckhoven C. Linkage and mutation analysis in an extended family with Charcot-Marie-Tooth disease type 1B. *J Med Genet* 1994; **31**: 811-815.

102. Mersiyanova IV, Ismailov SM, Polyakov AV, *et al.* Screening for mutations in the peripheral myelin genes PMP22, MPZ and Cx32 (GJB1) in Russian Charcot-Marie-Tooth neuropathy patients. *Hum Mutat* 2000; **15**: 340-347.

103. Lin KP, Soong BW, Chang MH, *et al.* Clinical and cellular characterization of two novel MPZ mutations, p.I135M and p.Q187PfsX63. *Clin Neurol Neurosurg* 2012; **114**: 124-129.

104. Roa BB, Warner LE, Garcia CA, *et al.* Myelin protein zero (MPZ) gene mutations in nonduplication type 1 Charcot-Marie-Tooth disease. *Hum Mutat* 1996; **7**: 36-45.

105. Prada V, Capponi S, Ursino G, *et al.* Sural nerve biopsy and functional studies support the pathogenic role of a novel MPZ mutation. *Neuropathology* 2015; **35**: 254-259.

106. Lerat J, Magdelaine C, Roux AF, *et al.* Hearing loss in inherited peripheral neuropathies: Molecular diagnosis by NGS in a French seires. *Mol Genet Genomic Med* 2019; **7**: e839.

107. Street VA, Meekins G, Lipe HP, *et al.* Charcot-Marie-Tooth neuropathy: clinical phenotypes of four novel mutations in the MPZ and Cx 32 genes. *Neuromuscul Disord* 2002; **12**: 643-650.

108. Leal A, Berghoff C, Berghoff M, *et al.* Charcot-Marie-Tooth disease: a novel Tyr145Ser mutation in the myelin protein zero (MPZ, P0) gene causes different phenotypes in homozygous and heterozygous carriers within one family. *Neurogenetics* 2003; **4**: 191-197.

109. Magot A, Latour P, Mussini JM, Mourtada R, Guiheneuc P, Pereon Y. A new MPZ mutation associated with a mild CMT1 phenotype presenting with recurrent nerve compression. *Muscle Nerve* 2008; **38**: 1055-1059.

110. Ohnishi A, Aoki A, Yamamoto T, Tsuji S. A case of Charcot-Marie-Tooth disease 1 B with Val 146Phe mutation of myelin protein zero showing a severe clinical phenotype. *Rinsho Shinkeigaku* 2000; **30**: 268-270.

111. Sun B, Chen Z, Ling L, Yang F, Huang X. Clinical and genetic spectra of Charcot-Marie-Tooth disease in Chinese Han patients. *J Peripher Nerv Syst* 2017; **22**: 13-18.

112. Lee H, Deignan JL, Dorrani N, *et al.* Clinical exome sequencing for genetic identification of rare Mendelian disorders. *JAMA* 2014; **312**: 1880-1887.

113. Eggers SDZ, Keswani SC, Melli G, Cornblath DR, *et al.* Clinical and genetic description of a family with Charcot-Marie-Tooth disease type 1B from a transmembrane MPZ mutation. *Muscle Nerve* 2004; **29**: 867-869.

114. van Doormaal TPC, van Ruissen F, Miller KJ, Hoogendijk JE, *et al.* Effective cauda equina decompression in two siblings with Charcot-Marie-Tooth disease type 1B. *Neuromuscul Disord* 2016; **26**: 837-840.

115. Simonati A, Fabrizi GM, Taioli F, Polo A, Cerini R, Rizzuto N, *et al.* Dejerine-Sottas neuropathy with multiple nerve roots enlargement and hypomyelination associated with a missense mutation of the transmembrane domain of MPZ/P0. *J Neurol* 2002; **249**: 1298-1302.

116. Banchs I, Casasnovas C, Montero J, Volpini V, Martínez-Matos JA. Charcot-Marie-Tooth disease with intermediate conduction velocities caused by a novel mutation in the MPZ gene. *Muscle Nerve* 2010; **42**:184-188.

117. Senderek J, Ramaekers VT, Zerres K, Rudnik-Schöneborn S, Schroder JM, Bergmann C. Phenotypic variation of a novel nonsense mutation in the P0 intracellular domain. *J Neurol Sci* 2001; **192**: 49-51.

118. Mandich P, Mancardi GL, Varese A, *et al.* Congenital hypomyelination due to myelin protein zero Q215X mutation. *Ann Neurol* 1999; **45**: 676-678.

119. Planté-Bordeneuve V, Parman Y, Guiochon-Mantel A, *et al.* The range of chronic demyelinating neuropathy of infancy: a clinico-pathological and genetic study of 15 unrelated cases. J Neurol 2001; **248**: 795-803.

120. Fabrizi GM, Pellegrini M, Angiari C, *et al.* Gene dosage sensitivity of a novel mutation in the intracellular domain of P0 associated with Charcot-Marie-Tooth disease type 1B. *Neuromuscul Disord 2006*; **16**: 183-187.

121. Schneider-Gold C, Kotting J, Epplen JT, Gold R, Gerding WM, *et al.* Unusual Charcot-Marie-Tooth phenotype due to a mutation within the intracellular domain of myelin protein zero. *Muscle Nerve* 2010; **41**: 550-554.

122. Shimizu H, Oka N, Kawarai T, *et al.* Late-onset CMT2 associated with a novel missense mutation in the cytoplasmic domain of the MPZ gene. *Clin Neurol Neurosurg* 2010; **112**:798-800.

123. Keckarevic-Markovic M, Milic-Rasic V, Mladenovic J, *et al.* Mutational analysis of GJB1, MPZ, PMP22, EGR2, and LITAF/SIMPLE in Serbian Charcot-Marie-Tooth patients. *J Peripher Nerv Syst* 2009; **14**: 125-136.

124. Crehalet H, Latour P, Bonnet V, *et al.* U1 snRNA mis-binding: a new cause of CMT1. *Neurogenetics* 2010; **11**: 13-19.

125. Corrado L, Magri S, Bagarotti A, *et al.* A novel synonymous mutation in the MPZ gene causing an aberrant splicing pattern and Charcot-Marie-Tooth disease type 1b. *Neuromuscul Disord* 2016; **26**: 516-520.

126. Lancaster E, Elman LB, Scherer SS, *et al.* A patient with neurofibromatosis type 1 and Charcot-Marie-Tooth disease type 1B. *Muscle Nerve* 2010; **41**: 555-558.

127. Bort S, Nelis E, Timmerman V, *et al.* Mutational analysis of the MPZ, PMP22 and Cx32 genes in patients of Spanish ancestry with Charcot-Marie-Tooth disease and hereditary neuropathy with liability to pressure palsies. *Hum Genet* 1997; **99**: 746-754.

128. Campagnolo M, Taioli F, Cacciavillan M, *et al.* Sporadic hereditary neuropathies misdiagnosed as chronic inflammatory demyelinating polyradiculoneuropathy: Pitfalls and red flags. *J Peripher Nerv Syst* 2020; **25**: 19-26.

129. Taioli F, Cabrini I, Cavallaro T, Simonati A, Testi S, Fabrizi GM. Déjerine-Sottas syndrome with a silent nucleotide change of myelin protein zero gene. *J Peripher Nerv Syst* 2011; **16**: 59-64.

130. Sabet A, Li J, Ghandour K, *et al.* Skin biopsies demonstrate MPZ splicing abnormalities in Charcot-Marie-Tooth neuropathy 1B. *Neurology* 2006; **67**: 1141-1146.

131. Chavada G, Rao DG, Martindale J, Hadjivassiliou M. A novel MPZ gene mutation in exon 2 causing late-onset demyelinating Charcot-Marie-Tooth disease. *J Clin Neuromuscul Dis* 2012; **13**: 206-208.

132. Ikegami T, Nicholson G, Ikeda H, *et al*. A novel homozygous mutation of the myelin Po gene producing Dejerine-Sottas disease (hereditary motor and sensory neuropathy type III). *Biochem Biophys Res Commun* 1996; **222**: 107-110.

133. Simpson BS, Rajabally YA, *et al.* Charcot-Marie-Tooth disease due to novel myelin protein zero mutation presenting as late-onset remitting sensory neuropathy. *J Clin Neuromuscul Dis* 2010; **11**: 187-190.

134. Kochański A, Kabzińska D, Drac H, Ryniewicz B, Rowinska-Marcinska K, Hausmanowa-Petrusewicz I. Early onset Charcot-Marie-Tooth type 1B disease caused by a novel Leu190fs mutation in the myelin protein zero gene. *Eur J Paediatr Neurol* 2004; **8**: 221-224.

135. Bellone E, Mandich P, James R, *et al.* Identification of a 4 bp deletion (1560del4) in P0 gene in a family with severe Charcot-Marie-Tooth disease. *Hum Mutat* 1996; **7**: 377-378.

136. Sivera R, Sevilla T, Vílchez JJ, *et al.* Charcot-Marie-Tooth disease: genetic and clinical spectrum in a Spanish clinical series. *Neurology* 2013; **81**: 1617-1625.

137. Ikegami T, Nicholson G, Ikeda H, *et al*. De novo mutation of the myelin Po gene in Déjérine-Sottas disease (hereditary motor and sensory neuropathy type III): two amino acid insertion after Asp 118. *Hum Mutat* 1998; **Suppl 1**: S103-105.

138. Piazza S, Baldinotti F, Fogli A, *et al.* A new truncating MPZ mutation associated with a very mild CMT1 B phenotype. *Neuromuscul Disord* 2010; **20**: 817-819.

139. Smit LS, Roofthooft D, van Ruissen F, Baas F, van Doorn PA. Congenital hypomyelinating neuropathy, a long term follow-up study in an affected family. *Neuromuscul Disord* 2008; **18**: 59-62.

140. Tachi N, Kozuka N, Ohya K, Chiba S, Yamashita S. A small direct tandem duplication of the myelin protein zero gene in a patient with Dejerine-Sottas disease phenotype. *J Neurol Sci* 1998; **156**:167-71.

141. Zschuntzsch J, Dibaj P, Pilgram S, Kotting J, Gerding WM, Neusch C. Severe demyelinating hypertrophic polyneuropathy caused by a de novo frameshift mutation within the intracellular domain of myelin protein zero (MPZ/P0). *J Neurol Sci* 2009; **281**: 113-115.

142. Rautenstrauss B, Nelis E, Grehl H, Pfeiffer RA, Van Broeckhoven C. Identification of a de novo insertional mutation in P0 in a patient with a Déjérine-Sottas syndrome (DSS) phenotype. *Hum Mol Genet* 1994; **3**: 1701-1702.

143. He J, Guo L, Xu G, *et al.* Clinical and genetic investigation in Chinese patients with demyelinating Charcot-Marie-Tooth disease. *J Peripher Nerv Syst* 2018; **23**: 216-226.

144. Silander K, Meretoja P, Nelis E, *et al.* A de novo duplication in 17p11.2 and a novel mutation in the Po gene in two Déjérine-Sottas syndrome patients. *Hum Mutat* 1996 ; **8**: 304-310.

145. Tyson J, Ellis D, Fairbrother U, et al. Hereditary demyelinating neuropathy of infancy. A genetically complex syndrome. *Brain* 1997; **120**: 47-63.

146. Szigeti K, Saifi GM, Armstrong D, Belmont JW, Miller G, Lupski JR. Disturbance of muscle fiber differentiation in congenital hypomyelinating neuropathy caused by a novel myelin protein zero mutation. *Ann Neurol* 2003; **54**: 398-402.

147. Pehlivan D, Beck CR, Okamoto Y, *et al.* The role of combined SNV and CNV burden in patients with distal symmetric polyneuropathy. *Genet Med* 2016; **18**: 443-451.

148. Maeda MH, Mitsui J, Soong BW, *et al.* Increased gene dosage of myelin protein zero causes Charcot-Marie-Tooth disease. *Ann Neurol* 2012; **71**: 84-92.

149. Speevak MD, Farrell SA, *et al.* Charcot-Marie-Tooth 1B caused by expansion of a familial myelin protein zero (MPZ) gene duplication. *Eur J Med Genet* 2013; **56**: 566-569.

150. Hoyer H, Braathen GJ, Eek AK, Skjelbred CF, Russell MB. Charcot-Marie-Tooth caused by a copy number variation in myelin protein zero. *Eur J Med Genet* 2011; **54**: e580-583.

151. Kerkhof J, Schenkel LC, Reilly J, *et al.* Clinical Validation of Copy Number Variant Detection from Targeted Next-Generation Sequencing Panels. *J Mol Diagn* 2017; **19**: 905-920.

152. Warner LE, Shohat M, Shorer Z, Lupski JR. Multiple de novo MPZ (P0) point mutations in a sporadic Dejerine-Sottas case. *Hum Mutat* 1997; **10**: 21-24.

153. Hayasaka K, Takada G, Ionasescu VV. Mutation of the myelin P0 gene in Charcot-Marie-Tooth neuropathy type 1B. *Hum Mol Genet* 1993; **2**: 1369-1372.

154, Hayasaka K, Himoro M, Sawaishi Y, et al. De novo mutation of the myelin P0 gene in Dejerine-Sottas disease (hereditary motor and sensory neuropathy type III). *Nat Genet* 1993; **5**: 266-268.

155. Blanquet-Grossard F, Pham-Dinh D, Dautigny A, et al. Charcot-Marie-Tooth type 1B neuropathy: third mutation of serine 63 codon in the major peripheral myelin glycoprotein P0 gene. *Clin Genet* 1995; **48**: 281-283.

156. Young T, Shuey N, Partridge J, Bremner FD, Nicholl DJ. Compound Charcot-Marie-Tooth disease: a kindred with severe hereditary neuropathy, pupil abnormalities and a novel MPZ mutation. *J Neurol Neurosurg Psychiatry* 2013; **84**: 234-236.

157. Himoro M, Yoshikawa H, Matsui T, *et al.* New mutation of the myelin P0 gene in a pedigree of Charcot-Marie-Tooth neuropathy 1. *Biochem Mol Biol Int* 1993; **31**: 169-173.

158. Hayasaka K, Himoro M, Sato W, *et al.* Charcot-Marie-Tooth neuropathy type 1B is associated with mutations of the myelin P0 gene. *Nat Genet* 1993; **5**: 31-34.

159. Latour P, Blanquet F, Nelis E, *et al.* Mutations in the myelin protein zero gene associated with Charcot-Marie-Tooth disease type 1B. *Hum Mutat* 1995; **6**: 50-54.

160. Lorance DK, Mandigo KA, Hehir MK. Novel Myelin Protein Zero Mutation in 3 Generations of Vermonters With Demyelinating Charcot-Marie-Tooth Disease. *J Clin Neuromuscul Dis* 2018; **19**: 101-107.

161. Ekici AB, Schweitzer D, Park O, *et al.* Charcot-Marie-Tooth disease and related peripheral neuropathies: novel mutations in the peripheral myelin genes connexin 32 (Cx32), peripheral myelin protein 22 (PMP22), and peripheral myelin protein zero (MPZ). *Neurogenetics* 2000; **3**: 49-50.

162. Nelis E, Van Broeckhoven C, De Jonghe P, *et al.* Estimation of the mutation frequencies in Charcot-Marie-Tooth disease type 1 and hereditary neuropathy with liability to pressure palsies: a European collaborative study. *Eur J Hum Genet* 1996; **4**: 25-33.

163. Kulkens T, Bolhuis PA, Wolterman RA, *et al.* Deletion of the serine 34 codon from the major peripheral myelin protein P0 gene in Charcot-Marie-Tooth disease type 1B. *Nat Genet* 1993; **5**: 35-39.

164. Simpson BS, Rajabally YA. Charcot-Marie-Tooth disease due to novel myelin protein zero mutation presenting as late-onset remitting sensory neuropathy. *J Clin Neuromuscul Dis* 2010; **11**: 187-90.
